# Immunomodulatory mechanisms of the gut microbiota and metabolites on regulatory T cells in rheumatoid arthritis

**DOI:** 10.3389/fimmu.2025.1610254

**Published:** 2025-07-07

**Authors:** Xuan Xu, Jingying Zhou, Haihua Xie, Ruhan Zhang, Bowen Gu, Li Liu, Mi Liu, Xiaorong Chang

**Affiliations:** ^1^ School of Acupuncture & Tuina and Rehabilitation, Hunan University of Chinese Medicine, Changsha, China; ^2^ The First Affiliated Hospital, Hunan University of Chinese Medicine, Changsha, China

**Keywords:** gut microbiota, metabolites, rheumatoid arthritis, regulatory T cells, autoimmune

## Abstract

Rheumatoid arthritis (RA) is an autoimmune disease, in which the abnormal activation and proliferation of effector T cells play a pivotal role in its pathogenesis. Regulatory T cells (Tregs) are a unique subset of immune cells with immunosuppressive functions, which help to inhibit the differentiation and proliferation of effector T cells in RA and maintain immune tolerance. The interaction between gut microbiota and immune cells has long been a research hotspot in autoimmune diseases. Although gut microbiota metabolites are considered to regulate the host’s immune system as a bridge of the gut-joint axis, how gut microbiota acts on immunosuppressive Tregs remains unclear. This review summarizes that how the gut microbiota directly or indirectly (via metabolites) enhances the immunosuppressive capacity of Tregs. This enhancement is primarily achieved through pathways such as promoting the induction of Tregs, upregulating the expression of characteristic transcription factors of Tregs, and facilitating their secretion of anti-inflammatory cytokines, thereby ameliorating the inflammatory microenvironment and subsequently improving autoimmune conditions in RA.

## 1 Introduction

Rheumatoid arthritis (RA) is a chronic systemic autoimmune disorder characterized by inflammatory arthritis presenting with symmetrical polyarticular pain and swelling (1). RA predominantly affects small joints in the hands and feet, potentially progressing to joint deformity and functional impairment, significantly impairing the patient’s quality of life and imposing a substantial burden on healthcare resources.

A growing body of research has demonstrated the crucial role of the gut microbiota in both the pathogenesis and therapeutic management of RA across clinical and animal models (2–4). Gut microbiota-derived metabolites serve as pivotal mediators of the gut-joint axis and play essential roles in maintaining intestinal barrier integrity, immune homeostasis, and modulating bone destruction in patients with RA (5). A recent Mendelian randomization study revealed that immune cells act as critical mediators of the intricate mechanisms linking gut microbiota to RA pathogenesis (6).

Regulatory T cells (Tregs), a distinct subset of lymphocytes with immunosuppressive properties, have emerged as promising therapeutic targets (7). Therefore, we aimed to review the mechanistic interplay between gut microbiota-derived metabolites and Tregs and systematically elucidate how microbial regulation enhances Treg-mediated immunosuppression to ameliorate RA progression.

## 2 Tregs

While most immune system cells function to promote inflammation to fight pathogens, Treg is a class of immune cells that can control immunity to maintain homeostasis (8). The currently accepted definition of Treg primarily involves its specific nuclear expression of the transcription factor FOXP3, along with surface-specific expression of CD25 (IL-2 receptor) and cytotoxic T lymphocyte-associated antigen 4 (CTLA-4). The discovery of the transcription factor FOXP3 represents a significant milestone, enabling the identification of Tregs within the CD4+ T cell subset.FOXP3 drives the differentiation of naïve CD4+ T cells into Tregs and serves as a master transcriptional regulator essential for Treg development and functional maintenance (9, 10). The establishment and maintenance of Treg functionality are strictly dependent on the stable, coordinated expression of lineage-specific genes, particularly FOXP3.

### 2.1 Treg classification

Based on their cellular origin, Tregs can be classified into three subtypes: thymus-derived natural Tregs (nTregs), peripherally induced Tregs (pTregs) originating from lymphoid organs, and *in vitro* differentiated induced Tregs (iTregs) generated from naïve T cells (11). Compared to other T-cell subsets, Treg cells exhibit an enhanced recognition capacity for self-antigens.

Tregs undergo antigen priming during thymic development (12). In the thymus, thymocytes with intermediate-affinity TCRs undergo differentiation upon receiving agonist signals from TCR/co-stimulatory molecules. Transforming growth factor-β (TGF-β) can induce FOXP3 expression and promote Treg cell development by disrupting weaker agonist signals (13). Upon thymic egress, Tregs express T-cell receptors (TCR) with high affinity for self-antigens (14). Tregs also demonstrate inhibitory functionality at peptide/MHC concentrations 100-fold lower than those required by conventional T-cell subsets (15). pTreg cells contribute to the maintenance of peripheral tolerance at inflammatory sites, particularly on mucosal surfaces such as the gut and lungs. Under inflammatory conditions, exogenous antigens derived from microbes can promote the generation of pTreg cells (16). However, studies have revealed that pTreg exhibits multiple significant characteristics independent of FOXP3 and can suppress the expansion of colonic effector T cells in a FOXP3-independent manner (17). This means FOXP3 is not the only gene required to maintain the development and function of Tregs. For instance, Helios can synergize with FOXP3 to enhance the function of Treg cells, augment the suppressive capacity of induced Treg cells, and upregulate the expression of various Treg cell-associated molecules (18, 19).

Additionally, there are also Treg cells that mediate immunosuppression without expressing FOXP3, such as Type 1 regulatory T cells (Tr1 cells), which are characterized by secreting IL-10 (20). Tr1 cells also possess immunosuppressive functions and can inhibit the expansion of pathogenic collagen-specific T cells in the collagen-induced arthritis (CIA) mouse model (21). Studies suggest that FOXP3+ Treg cells are indispensable during the initial phase of tolerance induction in inflammatory target organs, whereas Tr1 cells contribute to the maintenance of long-term tolerance (22). In addition to CTLA-4, Tregs also express various surface markers such as glucocorticoid-induced TNFR-related protein (GITR), latency-associated peptide (LAP), and/or lymphocyte-activation gene 3 (Lag-3), among others (23). The specific subsets of Tregs are classified based on cell surface or intracellular markers and their secreted cytokines, as shown in **Table 1**. Different subsets of Treg cells exhibit variations in the expression of chemokine receptors and transcription factors. There is no specific marker that can be used to distinguish all Tregs. However, the fact that some subgroups are similar, overcategorization may hinder the study of Treg. A comprehensive definition of human Tregs requires the integration of both phenotype and suppressive function (33). Although some Treg cell signature genes are indirectly regulated, most genes responsible for Treg cell stability and lineage determination are dependent on FOXP3 (34).

**Table 1:** Treg subgroup.

Subgroup name	Markers	Factors	References
FOXP3+Treg	CD25+ FOXP3+,Helios+ CTLA4+,Nrp1+,GITR	TGF-β,IL-10,IL-35	(24)
Tr1	FOXP3- CD49b+,LAG3+,CD226+,PD-1+,CTLA-4+	IL-10,TGF-β,IFN-r	(20)
Th3/Tr2	CD25+ CD69+,FOXP3- LAP+	TGF-β,IL-10	(25)
iTr35	FOXP3+,p35+	IL-35	(26)
B cell-derived Treg	CD25+ ,LAG3+,PD1v,ICOS+,CTLA4+,GITR+,CD49b+、FOXP3-、OX40+	IL-10,PD-1, CTLA4,LAG3,	(27)
T follicular regulatory (Tfr) cells	FOXP3+,CXCR5+,PD-1+,ICOS+	IL-10, TGF-b, and granzyme B	(28)
ExTreg (cytotoxic Treg)	CD16+,CD56+ CD25+,FOXP3+;	IL-17,IFN-y	(29)
Th1-like Treg	CD25+,FOXP3+,T-bet+,CXCR3+	IFN-γ	(30)
Th2-like Treg	CD25+, FOXP3+, CTLA-4+, GATA3+ CD44+, ICOS+, GITR+,	IL-4, IL-10;IL-5, IL-13	(31)
Th17-like Treg	CD25+,FOXP3+,RORγt+,CCR6+,CCR4,CD103+,ICOS+,Helios+	IL-10, TGF-β,IL-17A	(32)

### 2.2 Epigenetic modification

Epigenetic modifications are critical for Treg functionality, as they regulate the transcriptional control of Treg lineage-specific genes (24). The FOXP3 locus harbors three evolutionarily conserved non-coding sequences (CNS1-CNS3), and subsequent studies have identified an additional regulatory element (CNS0) positioned upstream of the transcriptional start site. These conserved regions contain dense clusters of hypomethylated CpG motifs that epigenetically regulate FOXP3 expression via DNA methylation-sensitive transcriptional modulation. CNS0 is epigenetically activated by the chromatin organizer SATB1 to initiate FOXP3 expression (35); CNS1 serves as a critical enhancer region mediating TGF-β-induced FOXP3 transcriptional activation (36); CNS3 is an indispensable regulatory element involved in FOXP3 induction; and CNS2 plays a non-redundant role in maintaining Treg lineage stability via sustained FOXP3 expression (37). IL-2 signaling induces STAT5 recruitment to the CNS2, driving Treg differentiation through chromatin remodeling (38).

The acetylation of FOXP3 is crucial for its functionality as it promotes the formation of FOXP3 dimers, enhances DNA-binding capacity, increases chromatin accessibility, and facilitates the interaction with transcription factors like FOXP3 (39). These processes regulate the immunosuppressive role of Treg cells. Histone acetyltransferases (HATs) and Histone deacetylase (HDACs) collectively maintain the dynamic equilibrium of acetylation in Treg cells. More than 10 HDAC enzymes are expressed in Treg cells. Histone deacetylase (HDAC) inhibition elevates histone acetylation at both the FOXP3 promoter and its cis-regulatory elements, potentiating Treg suppressive *in vitro* and *in vivo* functions (40).

## 3 Treg-mediated suppression of effector T-cell activation ameliorates RA

### 3.1 Central role of effector T cells in RA pathogenesis

Synovitis, a hallmark of RA, is histologically characterized by synovial hyperplasia, neovascularization, and heterogeneous inflammatory infiltrates, including lymphoid aggregates and germinal center-like structures (41). Infiltrating cells include T and B cells, macrophages, and dendritic cells. The pathogenesis of RA is not fully understood, but is primarily associated with immune dysregulation. The production of autoantibodies, including anti-citrullinated protein antibodies (ACPAs), and the presence of rheumatoid factor in both blood and synovial structures are considered prominent features of RA (42).

Autoimmune processes related to RA precede the onset of clinical disease by several years (43). Genetics is the most significant risk factor for the development of RA, with the HLA-DR locus facilitating the involvement of citrullinated antigens in CD4+ T-cell antigen presentation through shared epitopes. Binding to citrulline-modified peptides leads to the production of ACPAs, which further induces T-cell activation and cytokine production (34). Additionally, epigenetic factors, such as DNA methylation and histone acetylation, contribute to the pathogenesis of RA (44).

**Figure 1:**
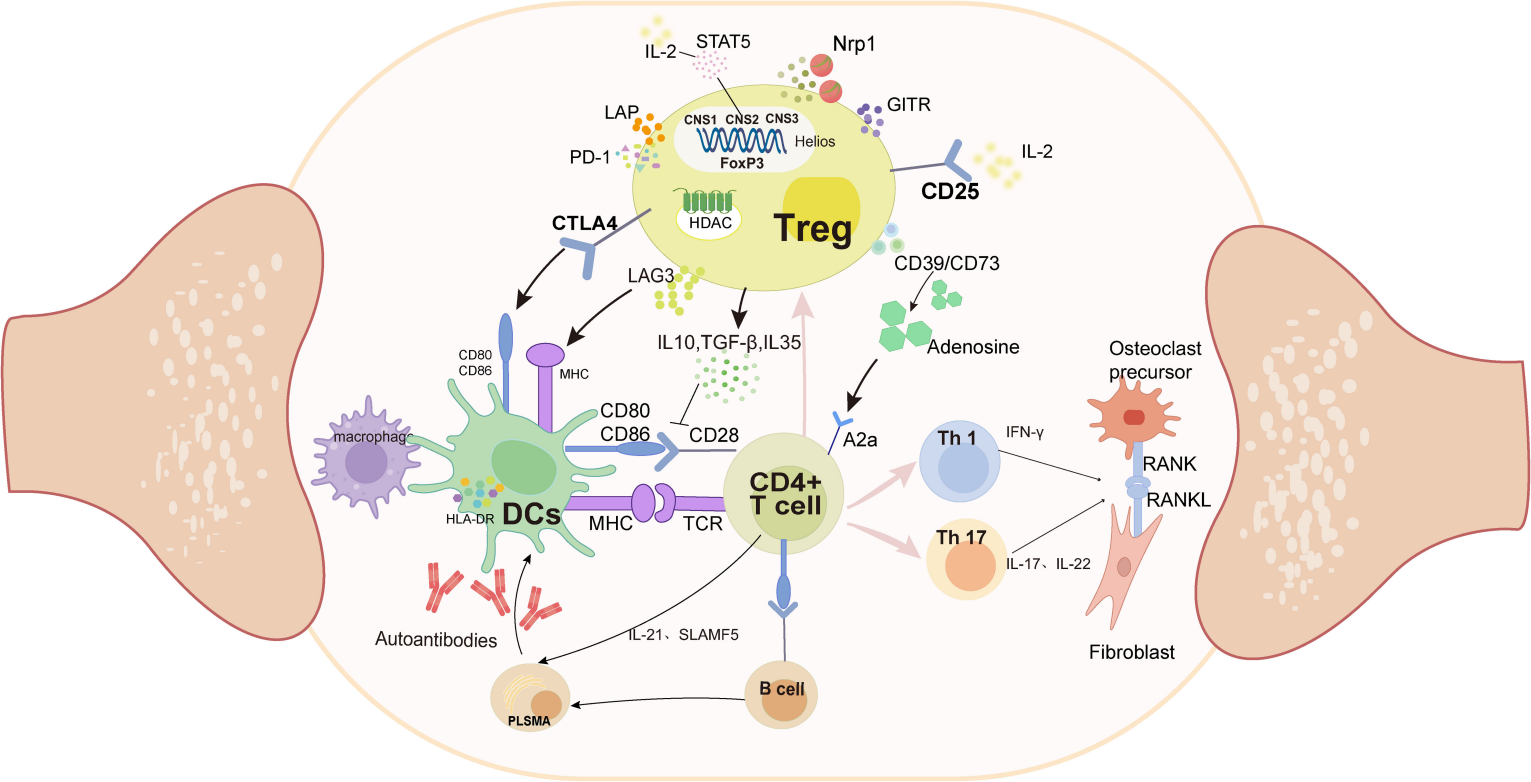
Pathologically, APCs (DCs and macrophages) present antigens to CD4+ T cells, which abnormally activate their differentiation into Th1 and Th17 subtypes. These two subtypes secrete pro-inflammatory factors such as IFN-γ and IL-17, which in turn lead to the proliferation of fibroblast-like synoviocytes, and ultimately bone destruction. In contrast, Treg cells inhibit abnormal antigen presentation by competitively binding to CD80/CD86 and MHC on the DCs surface; secreting anti-inflammatory factors and inhibiting Th proliferation through the CD39/CD73/A2a adenosine pathway. (DCs, Dendritic Cells;MHC, Major Histocompatibility Complex;TCR, T Cell Receptor; FOXP3, Foxhead Pox protein 3; HDAC, Histone deacetylase; CNS, conserved non-coding sequences; CTLA4, Cytotoxic T-Lymphocyte Associated protein 4;GITR, Glucocorticoid-induced TNFR-related protein; LAP, latency-associated peptide; Lag-3, lymphocyte-activation gene 3; PD-1, programmed cell death protein 1; NRP1, Neuropilin-1;RANK, receptor activator of NF- κB; RANKL, receptor activator of NF- κB ligand).

Cellular interactions within the synovium constitute a critical component of RA pathogenesis, with T cells playing a central role in the immune pathogenesis of rheumatoid arthritis (34) (**Figure 1**). Evaluation of a large array of cell lineages in synovial specimens from patients with RA have identified over 20 transcriptionally defined T-cell subtypes (45). Pathologically expanded peripheral T-helper cell subsets can induce plasma cell differentiation through IL-21 secretion and SLAMF5 interactions (46), subsequently leading to the production of ACPAs and other autoantibodies. These autoantibodies are then presented to T cells by antigen-presenting cells, including dendritic cells and macrophages, which activate and induce T-cell differentiation, along with the production of cytokines (47). In particular, helper T cells (Th cells), such as Th1 cells, promote inflammatory responses by secreting interferon-γ (IFN-γ), while Th17 cells exacerbate inflammation and tissue damage by producing cytokines like IL-17 and IL-22 (48). These cytokines, in turn, activate neighboring cells, including monocytes, macrophages, and synovial fibroblasts, to produce additional pro-inflammatory factors. These cytokines can stimulate the expression of RANKL, and the upregulation of RANKL further activates the NF-κB pathway, leading to the proliferation and activation of inflammatory cells, as well as the activation of osteoclasts. This leads to bone erosion and joint damage (49). In contrast, Tregs inhibit T-cell proliferation, activation, and effector function through multiple pathways.

### 3.2 Immunosuppressive mechanisms of Tregs in RA pathogenesis

Treg-mediated immunosuppression is primarily executed through three mechanistically distinct pathways: (1) regulatory cytokine secretion, (2) direct cell–cell contact-dependent suppression, and (3) metabolic modulation of target cells (50) (**Figure 1**). Treg cells elaborate upon an array of immunomodulatory cytokines critical for their suppressive functions, including interleukin-10 (IL-10), transforming growth factor-beta (TGF-β), and IL-35. IL-10 downregulates MHC-II and CD86 surface expression on dendritic cells and macrophages, impairing their antigen-presenting capacity to CD4+ T cells, thereby attenuating inflammatory responses in RA (51). TGF-β can inhibit T-cell proliferation, activation, and effector functions through dual mechanisms: downregulating dendritic cell function, interfering with antigen presentation, and concurrently inhibiting IL-2 production (52). IL-35 can inhibit Th17 differentiation and reduce the production of pro-inflammatory factors, thereby improving the inflammatory response in RA (53).

Cell–cell contact mediates immunosuppression. Dendritic cells (DCs) are the most effective antigen-presenting cells (APCs) in the immune system and possess bidirectional immune functions. While mature DCs activate effector T cells to drive inflammation, tolerogenic DCs upregulate anti-inflammatory cytokines (IL-10/TGF-β) with reduced co-stimulatory molecule expression, thereby promoting Treg differentiation and immunosuppression (54, 55). Treg surface-expressed CTLA-4 competitively antagonizes CD28 costimulation by binding to CD80/CD86 on DCs and transmitting inhibitory signals that suppress effector T-cell activation and clonal expansion (56). The transmembrane protein lymphocyte activation gene-3 (LAG-3) binds to MHC-II on APCs and inhibits DC activation through an ITAM-mediated inhibitory signaling pathway (57) and is currently emerging as an immunomodulatory target for various therapeutic approaches in RA (58). Furthermore, programmed cell death protein 1 (PD-1), a member of the CD28 superfamily, binds to its ligands, thereby inhibiting T-cell proliferation and IFN-γ production. PD-1 also plays a role in the regulation of osteoclast development in RA (59, 60).

Another mechanism of Treg cell-mediated suppression is disruption of target cell metabolism. CD39 and CD73 on the surface of Tregs act synergistically to produce adenosine, which binds to the adenosine receptor A2A on activated T cells, driving the inhibition of T-cell proliferation (61, 62). Studies have demonstrated the anti-inflammatory effects of adenosine on synovial cells in RA (63). Furthermore, the high expression levels of the IL-2 receptor (CD25) on the surface of Treg cells can bind to IL-2, reducing the activation and proliferation of effector T cells (64).

In RA pathogenesis, impairment of Treg migration and homeostasis may inhibit anti-inflammatory effects. IL-6-induced vasodilator-stimulated phosphoprotein phosphorylation inhibits Treg migration to inflammatory sites, thereby limiting their immunosuppressive functions (65). G protein signaling modulator 2 (GPSM2) is almost completely absent in RA, leading to abnormal G protein-coupled receptor signaling, which further weakens Treg migration (56). Additionally, abnormal expression of chemokines and adhesion molecules, such as CCR4, CCR6, and LFA-1 also affects the homing of Tregs to synovial tissue. A review summarized the existing research and concluded that strategies aimed at increasing the number of Tregs or enhancing their suppressive functions are effective in treating RA (7). Glucocorticoid drugs can upregulate FOXP3 expression to promote Treg expansion and enhance Treg function by modulating the TCR signaling pathway and cytokine milieu (66). Traditional antirheumatic drugs such as methotrexate (MTX) and cyclophosphamide (CTX) in combination therapy can enhance the suppressive function of Tregs (67). In addition to conventional drug therapies, low-dose IL-2 therapy has been demonstrated to alleviate symptoms by restoring Treg levels in RA patients (68). The underlying mechanism may involve selective expansion of Tregs through activation of the STAT5 signaling pathway (69).

## 4 The gut microbiota and RA

Abnormal immune responses at mucosal sites *in vivo* may be associated with the onset and progression of RA. The intestinal mucosa accounts for a significant portion of the mucosal content of the body, and microbial dysbiosis may interact with immune dysregulation, leading to more severe systemic immune disorders. The intestine is the largest immune organ in the human body and plays a crucial role in autoimmune diseases (70). The gut microbiota is a complex microbial ecosystem vital for maintaining host health and forming a mutually beneficial relationship. In the human body, the host provides a living environment for microorganisms that can supply nutrients and, more importantly, mount immune responses against pathogens to protect the body (71).

Researchers believe that the microbiota is crucial for various aspects of host immunity, including immune maturation, prevention of pathogen overgrowth, host cell proliferation and angiogenesis, regulation of intestinal physiological functions, and energy synthesis (72). Healthy gut microbiota is primarily characterized by diverse colonization, high microbial gene abundance, and stable core functions (73). Although its specific composition varies among individuals owing to factors such as genetics, age, diet, and environment, the functions of the adult gut microbiota remain relatively consistent in healthy individuals (74). In the gut, bacterial populations are predominantly anaerobic and include phyla such as Firmicutes and Bacteroidetes.

A dysbiotic gut microbiota is pathologically characterized by reduced microbial diversity, overrepresentation of specific taxa, compromised intestinal barrier integrity, and concomitant metabolic dysregulation (75). In clinical studies, significant changes in the gut microbiota composition have been observed in patients with RA. Xing et al. (76) found that both the Shannon diversity and Simpson index of gut bacteria were significantly lower in patients with RA than in healthy controls, indicating a reduction in prokaryotic diversity within samples under disease conditions and the presence of pronounced gut bacterial dysbiosis. At the phylum level, patients with RA exhibit a higher abundance of *Actinobacteria* and lower levels of *Bacteroidetes* and *Proteobacteria*. At the genus level, *Ruminococcus*, *Collinsella*, *Gemmiger*, and *Dorea* are significantly enriched, whereas *Bacteroides*, *Prevotella*, *Roseburia*, *Clostridium, Lactobacillus*, *Parabacteroides*, and *Megamonas* are markedly reduced. Similar alterations in the gut microbiota have been observed in animal studies. Compared to normal mice, a model group showed an increased relative abundance of *Lactobacillus*, *Candidatus_Arthromitus*, *UCG-005*, and *Anaerofustis*, whereas the relative abundance of *Bacteroides*, *Alistipes*, *Mucispirillum*, *unclassified_o_Bacteroidales*, and *norank_f:norank_o:Rhodospirillales* decreased (77). Due to the variety and large number of intestinal flora, and significant individual differences, coupled with the limitations of existing experimental methods, the results obtained from various experiments on flora changes are not completely consistent. More advanced technical means may need to be introduced to clarify the changes of flora. A Mendelian randomization study demonstrated that eight bacterial taxa were positively correlated with the risk of developing RA, whereas six taxa were negatively correlated with RA risk (78). Alterations in gut microbiota are highly evident in the pathogenesis of RA.

Ecological disturbances in the gut and autoimmune mechanisms may be involved in the development of RA (79). The interactions between mucosal sites and probiotic bacteria may play a role in the pathogenesis of RA (80). For instance, in the intestines of patients with RA, there is a reduction in certain beneficial bacteria, such as those from the *Bacteroidetes* phylum, and an increase in potentially pathogenic bacteria, such as *Prevotella* spp.; this imbalance in the microbiota may promote the development of RA by activating immune cells, fostering inflammatory responses, and affecting gut barrier function. An increase in *Lactobacillus* may drive joint inflammation by activating Th17 cells to secrete IL-17 (67), whereas a decrease in *Bacteroides* and *Alistipes* leads to insufficient differentiation of CD4+ T cells into Tregs (81). In addition, gut microbial dysbiosis can stimulate autoreactive cells to relocate to the joints, leading to inflammation (82).

Therefore, restoration of dysbiosis can alleviate inflammation levels. Bacteria in the gut can traverse the mucus layer and stimulate epithelial cells, thereby modulating immune function and enhancing the gut barrier (83). Zhao et al. found that Escherichia coli and Streptococcus bovis significantly influence levels of TNF-α and IL-6, with increased abundance of these bacteria enhancing ascorbic acid degradation, thereby mitigating the progression of RA (84). Gut microbiota can also interact directly with the host through microbial metabolites such as short-chain fatty acids (SCFAs) to regulate the immune system (85, 86). Through polysaccharide therapy, the gut microbiota breaks down polysaccharides into SCFAs (such as butyrate), which, by activating G-protein-coupled receptors (like GPR43), inhibit the NF-κB pathway and reduce the release of pro-inflammatory cytokines, thereby alleviating arthritis (77).

## 5 Gut microbiota and regulatory T cells

The intestine harbors a vast array of immune cells, including Tregs, which are abundant in the large intestine and are primarily represented as FOXP3+ Tregs (87). In the small intestine Tr1 cells are in relative abundance (88). Tregs are typically thought to be primed in the mesenteric lymph nodes before migrating to the gut. Additionally, FOXP3+ Tregs acquire secondary activation signals and expand in the small intestine to produce IL-10 (89). Intestinal epithelial cells are in close contact with immune cell populations, and secrete high levels of cytokines. For instance, IL-33 promotes the stability and function of Tregs (90) and modulates IL-18 secretion to help regulate CD4+ T cells in the colon, limiting their differentiation into Th17 cells, and enhancing the role of FOXP3+ Tregs in inflammation (91). The mucus layer above the intestinal epithelium is rich in large molecules called mucins. Upon inflammation, this layer can promote the production of IL-10 and TGF-β while reducing the production of pro-inflammatory cytokines, thereby facilitating Treg development (92). Intestinal mucosal dendritic cells can synergistically induce FOXP3+ Treg cells through the production of TGF-β and retinoic acid (93). Mice with specific knockout of TGF-β receptors exhibit reduced generation of FOXP3+ Tregs (94). Furthermore, microbial metabolites such as SCFAs can promote the development of peripheral Tregs (95). Intestinal microbiota can directly influence immune cells, and their metabolites affect immune cells. This complex interplay underscores the significance of the gut microbiota in shaping immune responses and maintaining immune homeostasis.

### 5.1 Bifidobacterium

*Bifidobacterium* is one of the most predominant physiological bacteria in the gastrointestinal tract of humans and animals and exerts its effects by reducing intestinal pH, inhibiting the growth of pathogens, maintaining the balance of intestinal flora, and preserving the integrity of the intestinal barrier (96). Bifidobacteria can alleviate RA by suppressing the production of IL-17 and other pro-inflammatory mediators (97). Furthermore, early administration of *bifidobacteria* can restore the healthy state of the intestinal flora in RA rat through histidine metabolism, thereby delaying the onset and progression of RA (98).

Treg cells serve as crucial mediators through which *Bifidobacterium* regulates the structure of intestinal flora. *Bifidobacterium* breve increases the expression of interleukin-10 receptor alpha (IL-10Rα) and the secretion of IL-10 in mouse Treg cells, forming an IL-10/IL-10Rα auto-stimulatory loop, thereby enhancing their immunosuppressive function (99). This process is associated with the enhanced mitochondrial activity of Tregs. Ravi Verma et al. (100) proposed that *Bifidobacterium >bifidum* is an effective inducer of FOXP3+ Tregs, with its cell surface glucan/galactan (CSGG) polysaccharide as a key component for Treg induction. CSGG activates intestinal dendritic cells (particularly CD103+CD11b+ DCs) through the TLR2 receptor, inducing their phenotypic transformation into regulatory dendritic cells (rDCs), which secrete IL-10 and TGF-β1, promoting the differentiation of naïve CD4+ T cells into Treg cells. This process is likely dependent on the MyD88 signaling pathway, a joint adapter protein within dendritic cells. A team from Jiangnan University discovered that *B. breve* CCFM1078 could alleviate joint inflammation in rat by modulating the intestinal flora and strengthening the intestinal barrier (101). Experimental results demonstrated that CCFM1078 increased the abundance of beneficial bacteria (such as *Bifidobacterium* spp. and *Faecalibaculum*), promoted the production of SCFAs, and enhanced the expression of FOXP3. Additionally, the genomic DNA of *B. longum* subsp. infantis significantly induces Tregs through unique methylated CpG motifs oligodeoxynucleotide (ODN) (102). CpG methylation is a crucial epigenetic mechanism that regulates Treg stability and function, promotes FOXP3 expression, enhances Treg differentiation, and bolsters their immunosuppressive capabilities. However, the latest research results show that ODN induces Treg most obviously at a certain dose, and excessive ODN will reduce the induction of Treg (103). It is therefore hypothesized that the effect of different levels of flora on Treg may also be different, which requires more research to verify.

*Bifidobacterium* can produce a variety of metabolites that are beneficial to the host, including vitamins, polyphenols, conjugated linoleic acids, and SCFAs. These metabolites, such as organic acids, bacteriocins, and biosurfactants, also exert detrimental effects on pathogenic microorganisms, thereby hindering the proliferation of harmful microbes (104). Yang et al. confirmed that *Bifidobacterium* can mitigate RA by suppressing inflammatory responses in CIA rat, regulating intestinal barrier function, and downregulating specific metabolites via the histidine metabolism pathway (98). Zhao et al. discovered that *Bifidobacterium* can mediate bile acid metabolism, wherein unconjugated bile acids can activate the TGR-5 receptor(, triggering the cAMP-PKA signaling pathway, which subsequently induces CREB to secrete IL-10 and promotes the Treg cell population in mouse (105).

### 5.2 Lactobacillus

*Lactobacillus* and other genera are widely used as probiotics, with intestinal glucose aiding their survival in acidic gastrointestinal environments (106). These probiotics alleviate RA through mechanisms such as immunomodulation, metabolite production, and improvement of intestinal barrier function (107, 108). *Lactobacillus* treatment can prevent the onset of arthritis in preclinical models, reduce arthritis scores in CIA rat and pro-inflammatory cytokines (such as IL-17, IL-1β, IL-6, and TNF-α), and increase the release of anti-inflammatory cytokines like IL-4 and IL-10 in bodily fluids (109). Fan et al. (110) clarified that *Lactobacillus casei* (particularly CCFM 1074) can alleviate symptoms in arthritic mice by modulating the Th17/Treg balance and enhancing the proportion of Treg cells in mesenteric lymph nodes. The process is primarily through local and systemic immune responses triggered by the production of SCFAs, which can induce the differentiation and proliferation of Treg cells. In rats with RA, following *Lactobacillus* casei intervention, symptoms were significantly alleviated, with restoration of intestinal flora homeostasis and regulation of oxidative stress balance. Further research revealed that *Lactobacillus casei* promotes the differentiation of CD4+ T cells into Treg cells by metabolizing tryptophan to produce indole derivatives that activate the aryl hydrocarbon receptor (AHR) on CD4+ T cells. *Lactobacillus* also maintains Tregs homeostasis through metabolites such as SCFAs (111).

*Lactobacillus rhamnosus* (LPR), another strain of Lactobacillus, promotes Treg differentiation of Tregs by forming a complex with secretory immunoglobulin A (SIgA) (112). This complex is taken up by DCs in Peyer’s patches (PPs), upregulating negative regulatory proteins of the Toll-like receptor (TLR) pathway (such as SIGIRR, Tollip, and SOCS1), thereby inhibiting the TLR signaling pathway and reducing inflammatory responses (113). Concurrently, the expression of retinaldehyde dehydrogenase 2 (RALDH2) is significantly enhanced, promoting the production of retinoic acid. Conversely, DCs in PP and mesenteric lymph nodes (MLN) secrete substantial amounts of IL-10 and TGF-β upon interaction with the LPR-SIgA complex. These DCs present antigens to naïve CD4+ T cells, and under the combined effects of IL-10, TGF-β, and retinoic acid, promote the differentiation of T cells into FOXP3+ Tregs (114). This entire process occurs within the PPs and MLN.

*Lactobacillus salivarius* is classified as a member of the genus *Ligilactobacillus* and is particularly notable for its immunomodulatory functions. *L. salivarius* has been isolated from patients with RA and has been found to elevate serum levels of IL-10 in arthritic mice, thereby increasing the proportion of Tregs (115). Additionally, *Lactobacillus salivarius* (especially strain FXJCJ7-2) can increase the proportion of FOXP3+ Tregs in the spleen, and its specific genes (such as LS_0679 and LS_0681) can boost the production of SCFAs (116). SCFAs (such as acetate and butyrate) directly promote Treg differentiation and mitigate inflammation by inhibiting the NF-κB pathway.

### 5.3 Bacteroides fragilis

*Bacteroides fragilis* can improve the symptoms of RA and enhance the efficacy of methotrexate (MTX) against arthritis in CIA rats by regulating butyrate metabolism (117). Moreover, *Bacteroides fragilis* produces a symbiotic factor known as polysaccharide A (PSA), which promotes mammalian immune system development. PSA facilitates the conversion of CD4+ T cells to FOXP3+ Tregs in germ-free mice, induces the expression of anti-inflammatory cytokines (such as IL-10 and TGF-β2), and enhances the suppressive function of Treg cells by directly activating the TLR2 signaling pathway on Treg cells (118). Subsequently, Telesford et al. demonstrated that PSA, mediated by DCs, induces the differentiation of naïve human CD4+ T cells into FOXP3+ Treg cells and significantly enhances the expression of CD39 on Treg cell surfaces (119). Upon activation by PSA, DCs upregulate molecules such as HLA-DR, CD86, CD40, and PD-L1, which promote the expression of FOXP3 and CD39 through direct contact with T cells.

### 5.4 Faecalibacterium prausnitzii

The abundance of *Faecalibacterium prausnitzii* is lower in patients with RA (120), whereas in healthy adults, *F. prausnitzii* constitutes 5–15% of intestinal bacteria (121). In a mouse model of RA, *F. prausnitzii* intervention reduced the levels of the pro-inflammatory cytokine IL-17, generated the metabolite butyrate, and improved the composition of the gut microbiota (122). Researchers identified a FOXP3-expressing, IL-10-secreting Treg subset in human colonic mucosa and blood, designated as DP8α based on its co-expression of CD4 and CD8α (123). This subset shares similarities with RORγt+ Treg, exhibiting a distinct hybrid profile of Tr1-like/cytotoxic CD4+ T cells. Besides demonstrating robust cytotoxicity, chemotaxis, and IgA-promoting capacity, the subset inhibits effector T-cell proliferation through CD39- and CD73-mediated purinergic metabolic pathways, thereby suppressing effector T-cell activity (124). The TCR on the surface of Tregs is more readily induced by *Faecalibacterium prausnitzii* within Clostridium cluster IV to trigger immunoregulatory responses (125). Additionally, this *bacterium* can induce dendritic cells to secrete anti-inflammatory cytokines IL-10 and IL-27 via the TLR2/TLR6 signaling pathway, differentiating into a “tolerogenic” phenotype that drives Tr1 cells differentiation and exerts immunosuppressive effects (126).

### 5.5 Prevotella

RA is associated with a relative expansion of the Prevotellaceae family in feces. Prior to the onset of RA, *Prevotella* species increased in individuals during the preclinical phase of RA (127). Colonization with *P. copri* exacerbates arthritis in a CIA model (128). However, unlike *P. copri*, *P. histicola* exhibits immunomodulatory properties and suppresses the production of inflammatory cytokines. Treatment with *P. histicola* significantly increased the number of CD103+ DCs in the intestinal lamina propria and spleen (129). These DCs possess immunomodulatory characteristics and can induce the differentiation of naïve CD4+ T cells into FOXP3+ regulatory T cells (Tregs) by secreting anti-inflammatory factors (such as IL-10) and expressing co-stimulatory molecules (such as CD80/86). Additionally, glucocorticoid-induced TNF receptor-related protein, which is highly expressed on the surface of Tregs, may maintain immune tolerance by inhibiting the mTOR pathway or promoting IL-10 secretion (130). A recent study found that P. histicola has a unique genetic sequence, and its unique outer membrane protein BtuB sequence may be the key to distinguishing it from pathogenic strains (131). The outer membrane protein is crucial in the interaction between bacteria and immune cells. The dual role of this flora reflects the complexity of intestinal microbial action. Due to the limitation of experimental level, it is not possible to identify and classify all strains, so more research is needed to clarify the classification of flora and its mechanism of action.

## 6 Gut microbial metabolites and Treg regulation

Intestinal microbes generate various metabolites through complex metabolic interactions that significantly affect various physiological processes within the host (132). In RA, gut microbiota metabolites can mediate communication between the commensal microbiota and the immune system, influencing the balance between anti-inflammatory Tregs and pro-inflammatory Th17 cell differentiation (133, 134).

### 6.1 Butyrate

SCFAs are primarily produced by specific subgroups of anaerobic bacteria, notably the members of the genera *Clostridium*, *Eubacterium*, and *Butyrivibrio*. Among these, *Clostridium* clusters IV and XIVa are efficient butyrate producers, with the dominant species *Faecalibacterium prausnitzii* and *Roseburia* spp., along with *Anaerostipes* and *Eubacterium* species, which play key roles (135). Additionally, dietary fiber serves as an appropriate substrate for the bacterial production of SCFAs (136).

Butyrate, a SCFA with anti-inflammatory and gut-barrier-regulating properties, serves as the primary energy source for colonocytes and plays a crucial role in the maintenance of intestinal homeostasis (137). The immunomodulatory effects on immune cells have attracted considerable attention. Butyrate can modulate T-cell differentiation and function while inhibiting the activation of myeloid cells such as dendritic cells, macrophages, and mast cells (138). As a canonical histone deacetylase (HDAC) inhibitor, butyrate enters cells either via passive diffusion or transmembrane proteins, directly binding to intracellular HDACs to inhibit their activity, or, at lower concentrations, is metabolized into acetyl-CoA to enhance histone acetylation (139). Specifically, butyrate upregulates FOXP3 expression by promoting histone H3 acetylation at the FOXP3 promoter and other conserved non-coding sequence regions within the FOXP3 locus, thereby inducing the differentiation of intestinal Tregs (140). Studies have suggested that butyrate-mediated Treg differentiation is dependent on the FOXP3 CNS1 enhancer (141). Da Som Kim et al. found that butyrate induces Treg expansion and production of the key anti-inflammatory factor IL-10 by inhibiting HDAC8 in T cells (142). Butyrate also induces differentiation of functional follicular regulatory T cells (TFR) directly *in vitro*, and also promotes histone hyperacetylation in the promoter region by inhibiting HDAC (143). On the other hand, butyrate enhances the differentiation and suppressive functions of FOXP3+ Tregs by activating GPR43 on Treg cells (144). Furthermore, butyrate, through a G protein-coupled receptor 43 (GPR43)-dependent mechanism, activates anti-inflammatory Treg cell responses in the colonic lamina propria. When the GPR43 gene was knocked out, the alleviation of inflammation diminished (145).

In addition to directly modulating Treg cell differentiation, butyrate also exhibits the capacity to regulate the expression of Treg cell-inducing molecules in epithelial cells or dendritic cells (DCs). For instance, it can stimulate epithelial cells to produce retinoic acid, a metabolite of dietary vitamin A, which serves as an essential cofactor for the generation of gut-specific Tregs by intestinal DCs (146, 147). Retinoic acid can induce the generation of antigen-specific Tr1 cells to prevent the occurrence and development of autoimmune diseases, and when combined with IL-2 it can enhance the induction (148). Furthermore, butyrate acts on DCs in the gut through its receptors, such as GPR109A and the butyrate transporter SLC5A8, to support DC-induced Treg cell differentiation (149).

Propionate and acetate are also natural HDAC inhibitors that can enhance lymphocyte activity and promote IL-10 production (150). However, their roles in RA remain poorly investigated. Studies have demonstrated that propionate alleviates disease symptoms in CIA mice, and *in vitro* experiments indicate that propionate upregulates FOXP3 gene expression and IL-10 production in T cells (151). Acetate has been found to facilitate the development and maturation of Treg cells in fetal mice (152), but its function in RA remains unexplored.

### 6.2 Bile acids

BA metabolism is mediated by bacteria with bile salt hydrolase activity, primarily *Lactobacillus*, *Bifidobacterium*, *Clostridium*, and *Bacteroides* (153, 154). These secondary bile acids (SBAs), generated through gut microbial metabolism, are involved in intestinal inflammatory responses (155). Numerous studies have elucidated the immunomodulatory properties of BAs within the gut (156–158). In RA models, BAs inhibit NLRP3 inflammasome activation and reduce the release of pro-inflammatory cytokines (such as IL-1β and IL-6) by binding to and activating the TGR5 receptor, thereby triggering the downstream cAMP-PKA signaling pathway (159).

BAs are signaling molecules that modulate immune homeostasis and exert control over host immune responses through a mechanism involving direct regulation of the balance between Th17 and Treg cells by BA metabolites. Primary BAs such as cholic acid and chenodeoxycholic acid (CDCA), are directly synthesized in the liver, whereas SBAs such as deoxycholic acid (DCA) and lithocholic acid (LCA), are generated via metabolic transformation by the gut microbiota. The positional arrangement of hydroxyl groups (α or β configuration) and the stereochemistry of the rings create structural variations in BAs, leading to the formation of diverse derivatives (160). Hang et al. screened over 30 BA metabolites and identified an LCA derivative, isoallolca, which enhanced Treg cell differentiation (161). Their findings revealed that isoallocalcin promotes Treg differentiation by augmenting mitochondrial reactive oxygen species (mitoROS) production, thereby increasing H3K27 acetylation in the FOXP3 promoter region. This process is regulated by the conserved non-coding enhancer CNS3 and requires TGF-β-induced signaling. Li et al. (156) further discovered that isoalloLCA increases the binding of the nuclear receptor NR4A1 to the FOXP3 locus, leading to enhanced FOXP3 gene transcription and subsequent Treg cell differentiation. Another DCA derivative, isoDCA, inhibits the immunostimulatory properties of dendritic cells (DCs) by antagonizing the activity of the farnesoid X receptor, shifting the balance toward immune tolerance and promoting the expansion of peripheral RORγt+ Treg cells in the colon. This process is dependent on CNS1 rather than on CNS3 (158).

### 6.3 Tryptophan

Tryptophan can be metabolized through two major pathways: kynurenine (Kyn) and indole. An imbalance in the kynurenine pathway, characterized by an increase in pro-inflammatory metabolites (such as QUIN) and a decrease in anti-inflammatory metabolites (such as KYNA and XANA), promotes synovial inflammation, cell proliferation, and joint destruction (162). Kynurenine is recognized as an endogenous ligand of the AhR and can activate AhR (163). Upon activation, AhR can facilitate the induction and differentiation of peripheral Tregs through the modulation of TGF-β and IL-2 signaling pathways (164). Tryptophan deficiency (such as excessive tryptophan consumption by IDO1/TDO enzymes in the tumor microenvironment) can directly trigger an increase in the transcription and protein expression of the AhR (165). Simultaneously, tryptophan depletion activates GCN2 kinase, which triggers the downstream transcription factor ATF4, subsequently upregulating amino acid transporter LAT1 (SLC7A5). This leads to a significant increase in kynurenine uptake, which further activates the AHR pathway. This dual sensitization enhances the induction of Tregs.

Furthermore, the indole pathway is highly dependent on the gut microbiota (166). Jiang et al. found that sinomenine treatment can elevate the levels of metabolites such as indole-3-acrylic acid (IA), indole-3-propionic acid (IPA), and indole-3-acetic acid (IAA) through the gut microbiota, thereby modulating tryptophan metabolism and activating AhR to regulate the Treg/Th17 balance, improving RA (167). *In vitro* experiments have also demonstrated that IAA can reduce the ubiquitination of FOXP3 through the AhR-TAZ-Tip60 pathway, promote the differentiation of Treg cells (168).

### 6.4 Other metabolites

Polyamine metabolites are abundant in the gut and can contribute to gut immunity (169). Spermidine, an antioxidant, can protect chondrocytes from IL-1β-mediated ferroptosis in rheumatoid arthritis (170). An *in vitro* experiment showed that spermidine can promote the differentiation of naive T cells into Tregs and upregulate the expression of FOXP3 (171). Furthermore, the gut microbiota produces hydrogen sulfide, a gaseous metabolite that enhances the expression of Tet1 and Tet2. This process catalyzes the conversion of 5-methylcytosine (5mC) to 5-hydroxymethylcytosine (5hmC) in FOXP3, establishing a Treg cell-specific hypomethylation pattern and stabilizing FOXP3 expression (172). However, the impact of these metabolites on Tregs has not been confirmed in RA animal models or clinical studies. These metabolites could potentially offer a promising avenue for future RA treatment.

## 7 Interactions among gut microbiota

There are interactions among microorganisms in the gut, which are dynamic and environment-dependent. These interactions encompass multiple aspects, including positive effects, such as cooperation, commensalism, and cross-feeding; and negative effects, like interference competition (173). Multiple clinical studies have found that probiotic supplements containing a mixture of various strains have improved the clinical symptoms of RA patients (174–176). These mixed strains are mainly Lactobacillus species. Further research found that the mixed strains of Lactobacillus acidophilus could restore the abundances of Lactobacillus and Clostridium in CIA rats and increase the content of the metabolite butyric acid (177). Moreover, the mixed strains were more effective than single strains. Ho - Keun Kwon et al. used a probiotic mixture of five strains, including Lactobacillus acidophilus, Lactobacillus casei, Lactobacillus reuteri, Bifidobacterium bifidum, and Streptococcus thermophilus, to intervene in RA mice. This intervention increased the level of FOXP3 in T cells, thereby increasing the number of CD4+FOXP3+ Tregs and inducing the regulatory dendritic cells (rDCs) to express high levels of IL - 10 and TGF - β, thus promoting the differentiation of FOXP3+Tregs (178). Such probiotics are the main sources of short-chain fatty acids and bile acids. The intervention of multiple strains may enhance the promotion of Tregs to improve RA. Therefore, determining the types and dosages of strains for combined use that can have a better effect on Treg cells and thus improve the therapeutic efficacy may be a direction for further research in the future.

Microbial metabolites also interact with each other in the gut. For example, bile acids and SCFAs may jointly participate in the regulation of cell proliferation through interrelated signaling pathways. They may have different effects at different concentrations. At high concentrations, SCFAs have anti - inflammatory effects, while secondary bile acids have pro - inflammatory effects (179). However, the interaction mechanism of multiple metabolites in the RA model has not been explored. More research designs are needed to investigate the network effects of co-existing metabolites.

## 8 Factors influencing the interaction between the gut microbiota and Tregs

### 8.1 Diet

Diet is considered the primary environmental factor influencing the gut microbiota. Therefore, diet-induced alterations in the microbiota can induce changes in host physiology (180). Fopei Ma et al. found that diet affects the inflammation of rheumatoid arthritis (RA) in mice through the circadian fluctuations of gut microbiota, and altering the diet timing reduces the anti - inflammatory effect (181). Teresina Laragione et al. found that a high-magnesium diet increased the number of Treg cells and alleviated the severity of arthritis in mice through an IL - 10-dependent manner mediated by gut microbiota (182). Among them, the level of Prevotella decreased, while the levels of bacteria such as Bacteroides increased.

In addition, a high-fiber diet rich in resistant starch (RS) increases the abundance of Bacteroides and circulating propionate, thereby promoting the increase of Treg cells and the production of IL-10 and improving arthritis in CIA mice (151). Ddietary fiber can also induce the biosynthesis of bile acids (183). The study summarizes that the Mediterranean diet rich in n-3 PUFAs, polyphenols, dietary fiber, and probiotics can regulate the gut microbiota. In particular, it can increase the content of short-chain fatty acids, reduce systemic inflammation, and improve the intestinal barrier function, thereby improving the prognosis of RA (184, 185).

Diet may promote the differentiation and function of Treg by enhancing the healthy metabolism of gut microbiota. Therefore, regular and healthy diet can restore the disrupted gut microbiota, regulate metabolism, and further promote the immunosuppressive function of Treg. However, another study found that the combination of a high-fiber diet and Prevotella could exacerbate the arthritis symptoms in RA mice, possibly because Prevotella leads to the overproduction of pro-inflammatory metabolites such as succinate (128). It is necessary to further determine that different doses and individual differences may yield different outcomes for the disease.

### 8.2 Other lifestyle

Exercise is considered to improve gut microbial health, and moderate aerobic exercise can increase the abundance of beneficial bacteria such as Bifidobacterium (186). Maintaining regular schedules and increasing sleep time may contribute to the stability of gut microbiota and gut health (187). Psychological stress can alter the composition of gut microbiota, reducing the abundance of some beneficial bacteria like Lactobacillus (188). Tobacco reduces the diversity of gut microbiota. Studies have shown that nicotine leads to an increase in Proteobacteria and Bacteroidetes and a decrease in Actinobacteria and Firmicutes in the mouse gut (189). Alcohol is also an important factor damaging the gut microbial environment. Research indicates that alcohol causes excessive growth of gut bacteria in mice, especially Enterobacteriaceae, thereby triggering gut inflammation (190). Therefore, a healthy lifestyle can enhance the abundance of beneficial bacteria, which are believed to promote the differentiation and function of Treg cells. However, further experimental studies are needed to clarify their correlation.

### 8.3 Drugs

Traditional anti - rheumatic drugs can regulate the gut microbiota to exert their effects (191). However, studies have found that most traditional anti - rheumatic drugs fail to increase the abundance of relevant flora and metabolites. After MTX intervention, the abundance of Bacteroides fragilis in mice decreased (192). Nevertheless, the absence of Bacteroides fragilis can make MTX treatment ineffective in CIA mice (117). This may be due to beneficial bacteria metabolizing to help drugs work.

Although antibiotics have been proven to reduce the abundance of RA-related pathogens (such as Prevotella copri) and short-term treatment can help alleviate inflammation (193), they may cause a decrease in gut microbiota diversity, changes in metabolic activities, and antibiotic resistance (194). Moreover, supplementing probiotics during antibiotic treatment cannot change the diversity index of the gut microbiome (195).

### 8.4 Chinese medicine

Traditional Chinese medicine can also regulate the immune system through the action of intestinal microorganisms to prevent RA (196). The herbal extract madecassoside promotes differentiation of Treg cells and expression of FOXP3 and IL-10 in mice by regulating intestinal flora and increasing butyric acid levels (197). Total Glucosides of Paeony up-regulated beneficial bacteria abundance and treg cell levels in CIA mice (198) [xx3]. Treatment with Fuzi increased bile acid content in CIA mice (199). In addition, the relative abundance of actinomycetes and lactobacillus in RA mice was significantly increased after acupuncture treatment, and such flora could increase the level of Treg cells (200).

## 9 Prospects for microbial therapy in RA

In the clinical treatment of RA, therapies targeting gut microbiota can help improve clinical symptoms in RA patients, including probiotic supplementation, prebiotics and fecal bacteria transplantation (134). But there are still a lot of challenges (1). Due to factors such as age, lifestyle, and eating habits, the intestinal flora changes dynamically and has significant individual differences (201), and the uncertainty about the differences between strains and doses makes it difficult to formulate standard personalized treatment plans (2). Probiotics are highly susceptible to environmental influences, and their stability in production, storage and transportation needs to be improved. However, the pharmaceutical development of butyrate faces challenges owing to its pungent odor, limited intestinal absorption, and rapid metabolic clearance as an energy substrate (202). To circumvent these limitations, serine-conjugated butyrate prodrugs have been developed via esterification. Experimental evidence demonstrated that this prodrug formulation enhances bioavailability while effectively modulating key immune cell populations in rat by reducing Th17 cell prevalence and promoting Treg differentiation (203) (3). The first-pass effect of liver and intestinal barrier limit the absorption and entry of metabolites into systemic circulation, and the chemical stability, hydrophilicity and lipophilicity of metabolites also affect systemic delivery efficiency. How to improve their bioavailability is the key issue to improve probiotic therapy (4). Due to the above reasons, the current clinical studies on intestinal microbial RA are of low quality. Most of them only observe their simple efficacy, which makes it difficult to deeply explore the true effect of intestinal microorganisms on Treg in human body. Therefore, there is insufficient evidence to prove its effectiveness and mechanism of action.

## 10 Conclusions and perspectives

**Figure 2:**
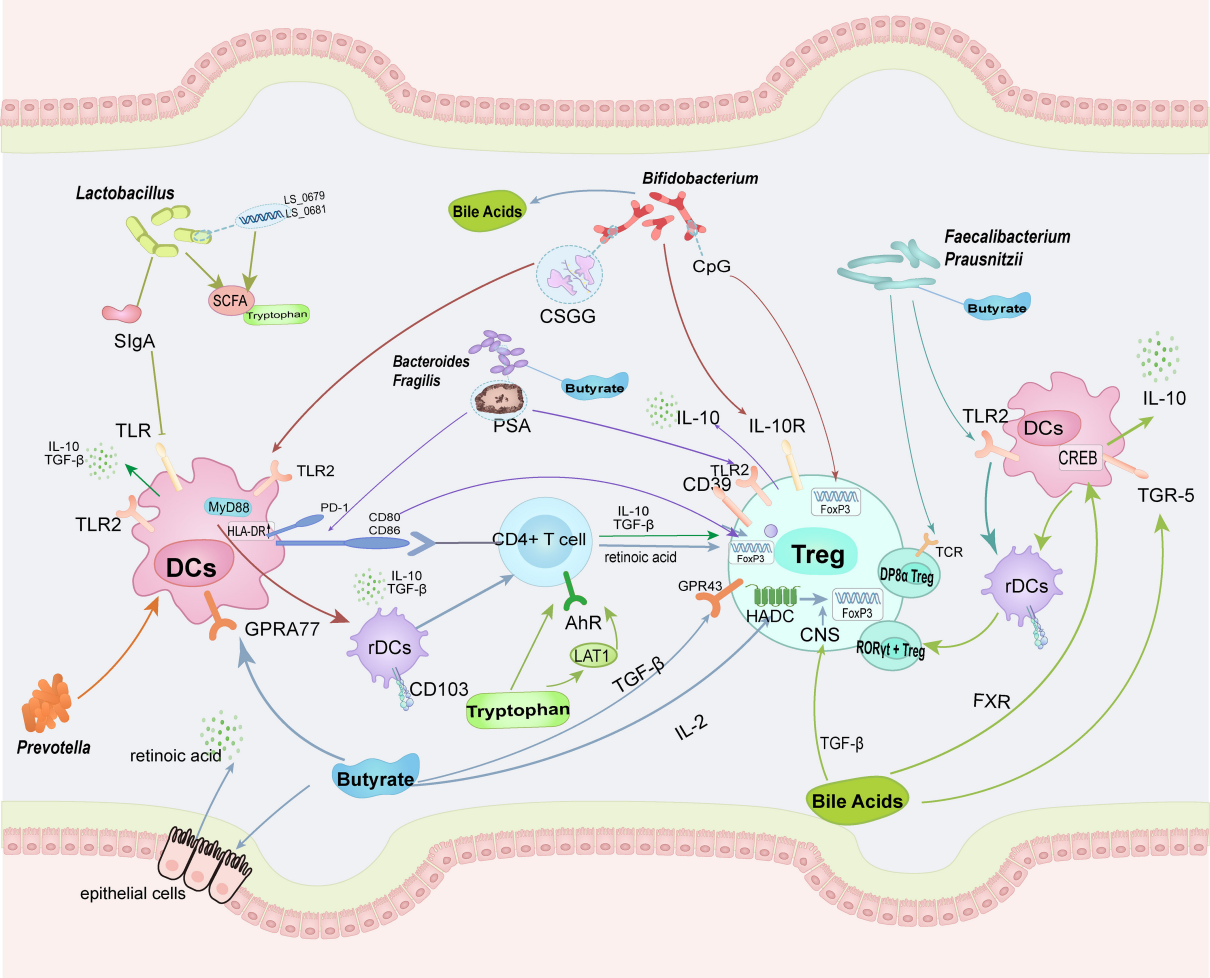
The intestinal microbiota and their metabolites can enhance the homeostasis and functionality of Tregs through direct cellular contact-mediated mechanisms. Concurrently, they promote the differentiation of regulatory DCs, which subsequently stimulate CD4+ T cells to preferentially differentiate into Tregs. Distinct arrow colors are utilized to delineate the differential regulatory pathways mediated by specific microbial taxa or metabolites. (TLR, Toll-like receptor; CSGG, cell surface glucan/galactan; PSA, polysaccharide A; PD-1, programmed cell death protein 1; GPR, G-protein-coupled receptors; TGR-5, Takeda G protein-coupled receptor 5; FXR, Farnesoid X Receptor).

**Figure 3:**
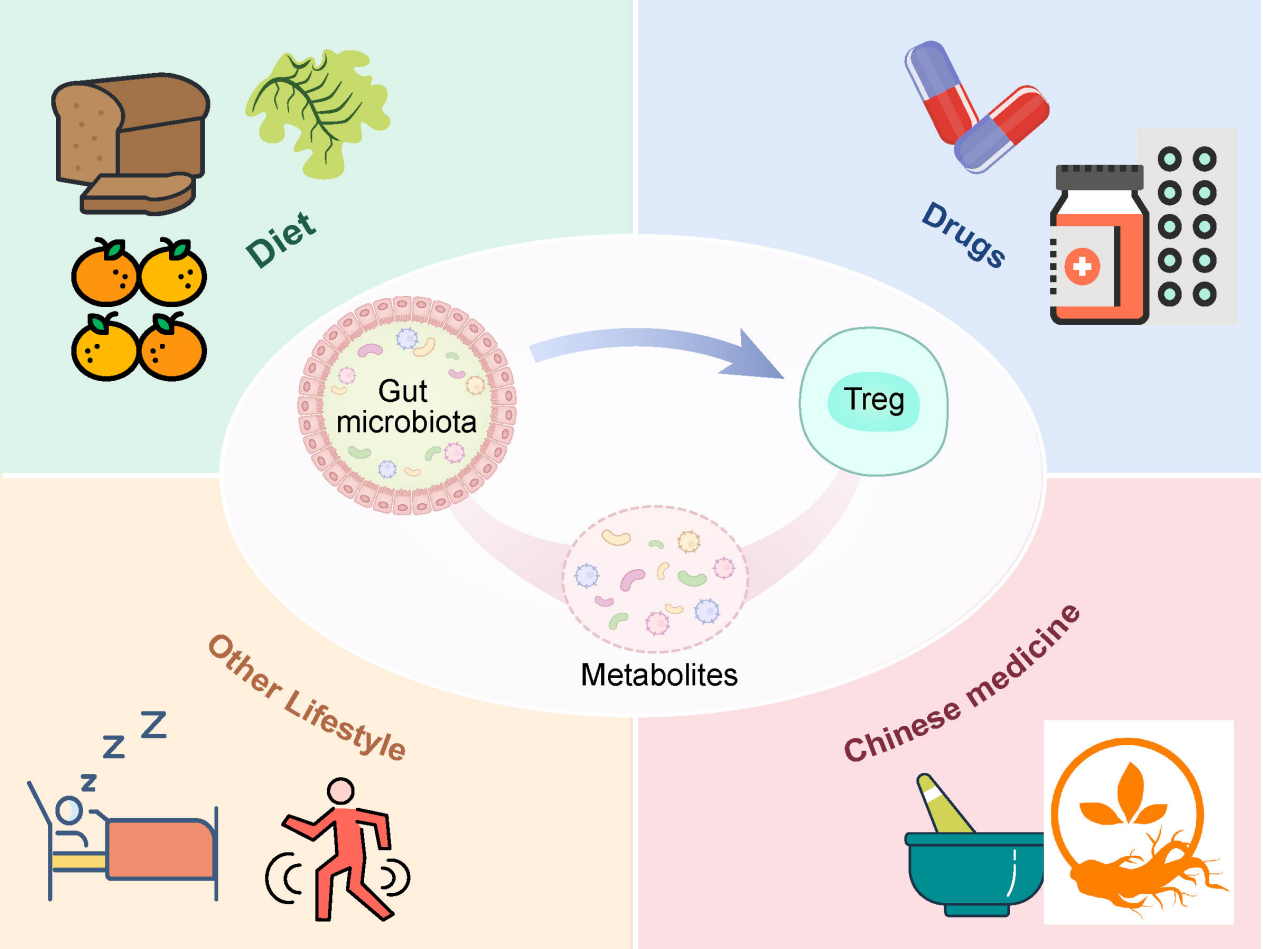
Factors influencing the interaction between the gut microbiota and Tregs.

Immunological imbalance is a critical mechanism underlying the pathogenesis of RA. Enhancing the induction of Tregs and maintaining their cellular function and homeostasis can ameliorate the immune imbalance in RA. The gut microbiota can enhance Treg induction and promote immunosuppressive functions. This process primarily occurs by promoting the differentiation of dendritic cells into tolerogenic DCs and inducing the differentiation of CD4+ T cells into Tregs. Increased secretion of anti-inflammatory factors during this process, especially IL-10, allows these beneficial bacteria to provide an anti-inflammatory environment for Treg differentiation and maturation. Additionally, the gut microbiota can directly or indirectly influence Treg surface proteins and intracellular transcription factors through metabolites, thereby strengthening the immunosuppressive pathways (**Figure 2**). In summary, modulation of the gut microbiota can regulate the overall immune environment, increase the secretion of anti-inflammatory factors, and help mitigate the overall inflammatory milieu. Multiple pathways further enhance the anti-inflammatory effects of Tregs, thereby improving the immune imbalance in RA. A healthy diet and lifestyle can improve symptoms by promoting the action of beneficial bacteria on Treg (**Figure 3**).

However, the gut microbiota comprises a wide variety of strains, and the microbiota composition varies significantly among individuals and exhibits dynamic changes. These factors limit in - depth exploration of the direct mechanism by which the microbiota acts on Treg. In the future, more in - vivo and in - vitro experiments are needed to explore more specific mechanisms of Treg, and these mechanisms should be verified in clinical research for translation into clinical practice.

## References

[1] Aletaha D Smolen JS. Diagnosis and management of rheumatoid arthritis: A review. Jama. (2018) 320:1360–72. doi: 10.1001/jama.2018.13103, PMID: 30285183

[2] Nii T Maeda Y Motooka D Naito M Matsumoto Y Ogawa T. Genomic repertoires linked with pathogenic potency of arthritogenic Prevotella copri isolated from the gut of patients with rheumatoid arthritis. Ann Rheum Dis. (2023) 82:621–29. doi: 10.1136/ard-2022-222881, PMID: 36627170 PMC10176341

[3] Gupta VK Cunningham KY Hur B Bakshi U Huang H Warrington KJ. Gut microbial determinants of clinically important improvement in patients with rheumatoid arthritis. Genome Med. (2021) 13:149. doi: 10.1186/s13073-021-00957-0, PMID: 34517888 PMC8439035

[4] Balasundaram D Veerasamy V Sylvia Singarayar M Neethirajan V Ananth Devanesan A Thilagar S. Therapeutic potential of probiotics in gut microbial homeostasis and Rheumatoid arthritis. Int Immunopharmacol. (2024) 137:112501. doi: 10.1016/j.intimp.2024.112501, PMID: 38885604

[5] Xu X Wang M Wang Z Chen Q Chen X Xu Y. The bridge of the gut-joint axis: Gut microbial metabolites in rheumatoid arthritis. Front Immunol. (2022) 13:1007610. doi: 10.3389/fimmu.2022.1007610, PMID: 36275747 PMC9583880

[6] Lan W Lu Q Ma W Jiang Z Chen Y Wang Z. Investigating the causal relationship between the gut microbiome and rheumatoid arthritis: mediating effects of immune cells. J Transl Med. (2025) 23:187. doi: 10.1186/s12967-025-06206-x, PMID: 39955590 PMC11830203

[7] Zhang J Liu H Chen Y Liu H Zhang S Yin G. Augmenting regulatory T cells: new therapeutic strategy for rheumatoid arthritis. Front Immunol. (2024) 15:1312919. doi: 10.3389/fimmu.2024.1312919, PMID: 38322264 PMC10844451

[8] Wardell CM Boardman DA Levings MK. Harnessing the biology of regulatory T cells to treat disease. Nat Rev Drug Discov. (2025) 24:93–111. doi: 10.1038/s41573-024-01089-x, PMID: 39681737

[9] Lu L Barbi J Pan F. The regulation of immune tolerance by FOXP3. Nat Rev Immunol. (2017) 17:703–17. doi: 10.1038/nri.2017.75, PMID: 28757603 PMC5793224

[10] Rudensky AY. Regulatory T cells and foxp3. Immunol Rev. (2011) 241:260–68. doi: 10.1111/j.1600-065X.2011.01018.x, PMID: 21488902 PMC3077798

[11] Campbell DJ Koch MA. Phenotypical and functional specialization of FOXP3+ regulatory T cells. Nat Rev Immunol. (2011) 11:119–30. doi: 10.1038/nri2916, PMID: 21267013 PMC3289970

[12] Takahashi T Sakaguchi S. Naturally arising CD25+CD4+ regulatory T cells in maintaining immunologic self-tolerance and preventing autoimmune disease. Curr Mol Med. (2003) 3:693–706. doi: 10.2174/1566524033479429, PMID: 14682491

[13] Tai X Indart A Rojano M Guo J Apenes N Kadakia T. How autoreactive thymocytes differentiate into regulatory versus effector CD4(+) T cells after avoiding clonal deletion. Nat Immunol. (2023) 24:637–51. doi: 10.1038/s41590-023-01469-2, PMID: 36959291 PMC10063450

[14] Wyss L Stadinski BD King CG Schallenberg S McCarthy NI Lee JY. Affinity for self antigen selects Treg cells with distinct functional properties. Nat Immunol. (2016) 17:1093–101. doi: 10.1038/ni.3522, PMID: 27478940 PMC4994872

[15] Takahashi T Kuniyasu Y Toda M Sakaguchi N Itoh M Iwata M. Immunologic self-tolerance maintained by CD25+CD4+ naturally anergic and suppressive T cells: induction of autoimmune disease by breaking their anergic/suppressive state. Int Immunol. (1998) 10:1969–80. doi: 10.1093/intimm/10.12.1969, PMID: 9885918

[16] Lathrop SK Bloom SM Rao SM Nutsch K Lio C Santacruz N. Peripheral education of the immune system by colonic commensal microbiota. Nature. (2011) 478:250–54. doi: 10.1038/nature10434, PMID: 21937990 PMC3192908

[17] van der Veeken J Campbell C Pritykin Y Schizas M Verter J Hu W. Genetic tracing reveals transcription factor Foxp3-dependent and Foxp3-independent functionality of peripherally induced Treg cells. Immunity. (2022) 55:1173–84. doi: 10.1016/j.immuni.2022.05.010, PMID: 35700740 PMC9885886

[18] Takatori H Kawashima H Matsuki A Meguro K Tanaka S Iwamoto T. Helios enhances treg cell function in cooperation with FoxP3. Arthritis Rheumatol. (2015) 67:1491–502. doi: 10.1002/art.39091, PMID: 25733061

[19] Ng MSF Roth TL Mendoza VF Marson A Burt TD. Helios enhances the preferential differentiation of human fetal CD4(+) naive T cells into regulatory T cells. Sci Immunol. (2019) 4(41):eaav5947. doi: 10.1126/sciimmunol.aav5947, PMID: 31757834 PMC7340007

[20] Vieira PL Christensen JR Minaee S O'Neill EJ Barrat FJ Boonstra A. IL-10-secreting regulatory T cells do not express Foxp3 but have comparable regulatory function to naturally occurring CD4+CD25+ regulatory T cells. J Immunol. (2004) 172:5986–93. doi: 10.4049/jimmunol.172.10.5986, PMID: 15128781

[21] Asnagli H Martire D Belmonte N Quentin J Bastian H Boucard-Jourdin M. Type 1 regulatory T cells specific for collagen type II as an efficient cell-based therapy in arthritis. Arthritis Res Ther. (2014) 16:R115. doi: 10.1186/ar4567, PMID: 24886976 PMC4075412

[22] Roncarolo MG Gregori S Bacchetta R Battaglia M Gagliani N. The biology of T regulatory type 1 cells and their therapeutic application in immune-mediated diseases. Immunity. (2018) 49:1004–19. doi: 10.1016/j.immuni.2018.12.001, PMID: 30566879

[23] Mason GM Lowe K Melchiotti R Ellis R de Rinaldis E Peakman M. Phenotypic complexity of the human regulatory T cell compartment revealed by mass cytometry. J Immunol. (2015) 195:2030–37. doi: 10.4049/jimmunol.1500703, PMID: 26223658

[24] Sumida TS Cheru NT Hafler DA. The regulation and differentiation of regulatory T cells and their dysfunction in autoimmune diseases. Nat Rev Immunol. (2024) 24:503–17. doi: 10.1038/s41577-024-00994-x, PMID: 38374298 PMC11216899

[25] Gandhi R Farez MF Wang Y Kozoriz D Quintana FJ Weiner HL. Cutting edge: human latency-associated peptide+ T cells: a novel regulatory T cell subset. J Immunol. (2010) 184:4620–24. doi: 10.4049/jimmunol.0903329, PMID: 20368276 PMC2904991

[26] Collison LW Chaturvedi V Henderson AL Giacomin PR Guy C Bankoti J. IL-35-mediated induction of a potent regulatory T cell population. Nat Immunol. (2010) 11:1093–101. doi: 10.1038/ni.1952, PMID: 20953201 PMC3008395

[27] Chien C Chiang B. Regulatory T cells induced by B cells: a novel subpopulation of regulatory T cells. J BioMed Sci. (2017) 24:86. doi: 10.1186/s12929-017-0391-3, PMID: 29151021 PMC5694621

[28] Fonseca VR Ribeiro F Graca L. T follicular regulatory (Tfr) cells: Dissecting the complexity of Tfr-cell compartments. Immunol Rev. (2019) 288:112–27. doi: 10.1111/imr.12739, PMID: 30874344

[29] Freuchet A Roy P Armstrong SS Oliaeimotlagh M Kumar S Orecchioni M. Identification of human exT(reg) cells as CD16(+)CD56(+) cytotoxic CD4(+) T cells. Nat Immunol. (2023) 24:1748–61. doi: 10.1038/s41590-023-01589-9, PMID: 37563308 PMC11022744

[30] Zheng J Liu Y Qin G Lam KT Guan J Xiang Z. Generation of human Th1-like regulatory CD4+ T cells by an intrinsic IFN-γ- and T-bet-dependent pathway. Eur J Immunol. (2011) 41:128–39. doi: 10.1002/eji.201040724, PMID: 21182084

[31] Jin H Park Y Elly C Liu Y. Itch expression by Treg cells controls Th2 inflammatory responses. J Clin Invest. (2013) 123:4923–34. doi: 10.1172/JCI69355, PMID: 24135136 PMC3809788

[32] Yang B Hagemann S Mamareli P Lauer U Hoffmann U Beckstette M. Foxp3(+) T cells expressing RORgammat represent a stable regulatory T-cell effector lineage with enhanced suppressive capacity during intestinal inflammation. Mucosal Immunol. (2016) 9:444–57. doi: 10.1038/mi.2015.74, PMID: 26307665

[33] Gootjes C Zwaginga JJ Roep BO Nikolic T. Defining human regulatory T cells beyond FOXP3: the need to combine phenotype with function. Cells. (2024) 13:941. doi: 10.3390/cells13110941, PMID: 38891073 PMC11172350

[34] Ramirez RN Chowdhary K Leon J Mathis D Benoist C. FoxP3 associates with enhancer-promoter loops to regulate T(reg)-specific gene expression. Sci Immunol. (2022) 7:eabj9836. doi: 10.1126/sciimmunol.abj9836, PMID: 35030035 PMC9059705

[35] Kitagawa Y Ohkura N Kidani Y Vandenbon A Hirota K Kawakami R. Guidance of regulatory T cell development by Satb1-dependent super-enhancer establishment. Nat Immunol. (2017) 18:173–83. doi: 10.1038/ni.3646, PMID: 27992401 PMC5582804

[36] Tone Y Furuuchi K Kojima Y Tykocinski ML Greene MI Tone M. Smad3 and NFAT cooperate to induce Foxp3 expression through its enhancer. Nat Immunol. (2008) 9:194–202. doi: 10.1038/ni1549, PMID: 18157133

[37] Chen Q Kim YC Laurence A Punkosdy GA Shevach EM. IL-2 controls the stability of Foxp3 expression in TGF-beta-induced Foxp3+ T cells. vivo. J Immunol. (2011) 186:6329–37. doi: 10.4049/jimmunol.1100061, PMID: 21525380 PMC3098943

[38] Feng Y Arvey A Chinen T van der Veeken J Gasteiger G Rudensky AY. Control of the inheritance of regulatory T cell identity by a cis element in the Foxp3 locus. Cell. (2014) 158:749–63. doi: 10.1016/j.cell.2014.07.031, PMID: 25126783 PMC4151558

[39] Wang L Beier UH Akimova T Dahiya S Han R Samanta A. Histone/protein deacetylase inhibitor therapy for enhancement of Foxp3+ T-regulatory cell function posttransplantation. Am J Transplant. (2018) 18:1596–603. doi: 10.1111/ajt.14749, PMID: 29603600 PMC6035084

[40] Tao R de Zoeten EF Ozkaynak E Chen C Wang L Porrett PM. Deacetylase inhibition promotes the generation and function of regulatory T cells. Nat Med. (2007) 13:1299–307. doi: 10.1038/nm1652, PMID: 17922010

[41] Gravallese EM Firestein GS. Rheumatoid arthritis - common origins, divergent mechanisms. N Engl J Med. (2023) 388:529–42. doi: 10.1056/NEJMra2103726, PMID: 36780677

[42] Di Matteo A Bathon JM Emery P. Rheumatoid arthritis. Lancet. (2023) 402:2019–33. doi: 10.1016/S0140-6736(23)01525-8, PMID: 38240831

[43] Mankia K Emery P. Preclinical rheumatoid arthritis: progress toward prevention. Arthritis Rheumatol. (2016) 68:779–88. doi: 10.1002/art.39603, PMID: 26814677

[44] Yang C Li D Teng D Zhou Y Zhang L Zhong Z. Epigenetic regulation in the pathogenesis of rheumatoid arthritis. Front Immunol. (2022) 13:859400. doi: 10.3389/fimmu.2022.859400, PMID: 35401513 PMC8989414

[45] Zhang F Wei K Slowikowski K Fonseka CY Rao DA Kelly S. Defining inflammatory cell states in rheumatoid arthritis joint synovial tissues by integrating single-cell transcriptomics and mass cytometry. Nat Immunol. (2019) 20:928–42. doi: 10.1038/s41590-019-0378-1, PMID: 31061532 PMC6602051

[46] Rao DA Gurish MF Marshall JL Slowikowski K Fonseka CY Liu Y. Pathologically expanded peripheral T helper cell subset drives B cells in rheumatoid arthritis. Nature. (2017) 542:110–14. doi: 10.1038/nature20810, PMID: 28150777 PMC5349321

[47] Kedmi R Littman DR. Antigen-presenting cells as specialized drivers of intestinal T cell functions. Immunity. (2024) 57:2269–79. doi: 10.1016/j.immuni.2024.09.011, PMID: 39383844

[48] Sakuragi T Yamada H Haraguchi A Kai K Fukushi J Ikemura S. Autoreactivity of peripheral helper T cells in the joints of rheumatoid arthritis. J Immunol. (2021) 206:2045–51. doi: 10.4049/jimmunol.2000783, PMID: 33846228

[49] Ilchovska DD Barrow DM. An Overview of the NF-kB mechanism of pathophysiology in rheumatoid arthritis, investigation of the NF-kB ligand RANKL and related nutritional interventions. Autoimmun Rev. (2021) 20:102741. doi: 10.1016/j.autrev.2020.102741, PMID: 33340772

[50] Yan S Kotschenreuther K Deng S Kofler DM. Regulatory T cells in rheumatoid arthritis: functions, development, regulation, and therapeutic potential. Cell Mol Life Sci. (2022) 79:533. doi: 10.1007/s00018-022-04563-0, PMID: 36173485 PMC9522664

[51] Yuba E Budina E Katsumata K Ishihara A Mansurov A Alpar AT. Suppression of rheumatoid arthritis by enhanced lymph node trafficking of engineered interleukin-10 in murine models. Arthritis Rheumatol. (2021) 73:769–78. doi: 10.1002/art.41585, PMID: 33169522 PMC11095083

[52] Larson C Oronsky B Carter CA Oronsky A Knox SJ Sher D. TGF-beta: a master immune regulator. Expert Opin Ther Targets. (2020) 24:427–38. doi: 10.1080/14728222.2020.1744568, PMID: 32228232

[53] Xin PL Jie LF Cheng Q Bin DY Dan CW. Pathogenesis and function of interleukin-35 in rheumatoid arthritis. Front Pharmacol. (2021) 12:655114. doi: 10.3389/fphar.2021.655114, PMID: 34054534 PMC8155723

[54] Raker VK Domogalla MP Steinbrink K. Tolerogenic dendritic cells for regulatory T cell induction in man. Front Immunol. (2015) 6:569. doi: 10.3389/fimmu.2015.00569, PMID: 26617604 PMC4638142

[55] Cai Z Zhang W Yang F Yu L Yu Z Pan J. Immunosuppressive exosomes from TGF-beta1 gene-modified dendritic cells attenuate Th17-mediated inflammatory autoimmune disease by inducing regulatory T cells. Cell Res. (2012) 22:607–10. doi: 10.1038/cr.2011.196, PMID: 22157651 PMC3292292

[56] Tekguc M Wing JB Osaki M Long J Sakaguchi S. Treg-expressed CTLA-4 depletes CD80/CD86 by trogocytosis, releasing free PD-L1 on antigen-presenting cells. Proc Natl Acad Sci U.S.A. (2021) 118:e2023739118. doi: 10.1073/pnas.2023739118, PMID: 34301886 PMC8325248

[57] Wakamatsu E Machiyama H Toyota H Takeuchi A Hashimoto R Kozono H. Indirect suppression of CD4 T cell activation through LAG-3-mediated trans-endocytosis of MHC class II. Cell Rep. (2024) 43:114655. doi: 10.1016/j.celrep.2024.114655, PMID: 39191259

[58] Gertel S Polachek A Eviatar T Elkayam O Furer V. A novel inhibitory pathway of synovial inflammation exerted by glucocorticoids and tumour necrosis factor inhibitors via lymphocyte activation gene-3 up-regulation: an ex vivo study. Rheumatol (Oxford). (2025) 64:1689–97. doi: 10.1093/rheumatology/keae389, PMID: 39052869

[59] Zhulai G Oleinik E. Targeting regulatory T cells in anti-PD-1/PD-L1 cancer immunotherapy. Scand J Immunol. (2022) 95:e13129. doi: 10.1111/sji.13129, PMID: 34936125

[60] Hu Q Yuan Y Li Y Yang L Zhou X Xiong D. Programmed cell death ligand 1-transfected mouse bone marrow mesenchymal stem cells as targeted therapy for rheumatoid arthritis. BioMed Res Int. (2021) 2021:5574282. doi: 10.1155/2021/5574282, PMID: 34497850 PMC8421163

[61] Romio M Reinbeck B Bongardt S Huls S Burghoff S Schrader J. Extracellular purine metabolism and signaling of CD73-derived adenosine in murine Treg and Teff cells. Am J Physiol Cell Physiol. (2011) 301:C530–39. doi: 10.1152/ajpcell.00385.2010, PMID: 21593451

[62] Bopp T Becker C Klein M Klein-Hessling S Palmetshofer A Serfling E. Cyclic adenosine monophosphate is a key component of regulatory T cell-mediated suppression. J Exp Med. (2007) 204:1303–10. doi: 10.1084/jem.20062129, PMID: 17502663 PMC2118605

[63] Sohn R Junker M Meurer A Zaucke F Straub RH Jenei-Lanzl Z. Anti-inflammatory effects of endogenously released adenosine in synovial cells of osteoarthritis and rheumatoid arthritis patients. Int J Mol Sci. (2021) 22:8956. doi: 10.3390/ijms22168956, PMID: 34445661 PMC8396606

[64] de la Rosa M Rutz S Dorninger H Scheffold A. Interleukin-2 is essential for CD4+CD25+ regulatory T cell function. Eur J Immunol. (2004) 34:2480–88. doi: 10.1002/eji.200425274, PMID: 15307180

[65] Kotschenreuther K Yan S Kofler DM. Migration and homeostasis of regulatory T cells in rheumatoid arthritis. Front Immunol. (2022) 13:947636. doi: 10.3389/fimmu.2022.947636, PMID: 36016949 PMC9398455

[66] Bereshchenko O Coppo M Bruscoli S Biagioli M Cimino M Frammartino T. GILZ promotes production of peripherally induced Treg cells and mediates the crosstalk between glucocorticoids and TGF-beta signaling. Cell Rep. (2014) 7:464–75. doi: 10.1016/j.celrep.2014.03.004, PMID: 24703841

[67] Yu X Wang C Luo J Zhao X Wang L Li X. Combination with methotrexate and cyclophosphamide attenuated maturation of dendritic cells: inducing Treg skewing and Th17 suppression. vivo. Clin Dev Immunol. (2013) 2013:238035. doi: 10.1155/2013/238035, PMID: 24194771 PMC3806152

[68] Wang J Zhang S Chang J Cheng T Jiang X Su Q. Low-dose IL-2 improved clinical symptoms by restoring reduced regulatory T cells in patients with refractory rheumatoid arthritis: A randomized controlled trial. Front Immunol. (2022) 13:947341. doi: 10.3389/fimmu.2022.947341, PMID: 36524114 PMC9744779

[69] Zhou P. Emerging mechanisms and applications of low-dose IL-2 therapy in autoimmunity. Cytokine Growth Factor Rev. (2022) 67:80–88. doi: 10.1016/j.cytogfr.2022.06.003, PMID: 35803833

[70] Wang S Liu Y Zou X Pan M Wan Q Chu X. Exploring the pathogenesis of RA through the gut-articular axis-dysbiosis a potential factor. Clin Anat. (2025) 38:134–45. doi: 10.1002/ca.24215, PMID: 39189295

[71] Zegarra-Ruiz DF Kim DV Norwood K Kim M Wu WH Saldana-Morales FB. Thymic development of gut-microbiota-specific T cells. Nature. (2021) 594:413–17. doi: 10.1038/s41586-021-03531-1, PMID: 33981034 PMC8323488

[72] Lynch SV Pedersen O. The human intestinal microbiome in health and disease. N Engl J Med. (2016) 375:2369–79. doi: 10.1056/NEJMra1600266, PMID: 27974040

[73] Van Hul M Cani PD Petitfils C De Vos WM Tilg H El-Omar EM. What defines a healthy gut microbiome? Gut. (2024) 73:1893–908. doi: 10.1136/gutjnl-2024-333378, PMID: 39322314 PMC11503168

[74] Joos R Boucher K Lavelle A Arumugam M Blaser MJ Claesson MJ. Examining the healthy human microbiome concept. Nat Rev Microbiol. (2025) 23:192–205. doi: 10.1038/s41579-024-01107-0, PMID: 39443812

[75] Wang H Cai Y Wu W Zhang M Dai Y Wang Q. Exploring the role of gut microbiome in autoimmune diseases: A comprehensive review. Autoimmun Rev. (2024) 23:103654. doi: 10.1016/j.autrev.2024.103654, PMID: 39384149

[76] Xing Y Liu Y Sha S Zhang Y Dou Y Liu C. Multikingdom characterization of gut microbiota in patients with rheumatoid arthritis and rheumatoid arthritis-associated interstitial lung disease. J Med Virol. (2024) 96:e29781. doi: 10.1002/jmv.29781, PMID: 38961767

[77] Dong Y Wang Y Zhang F Ma J Li M Liu W. Polysaccharides from Gaultheria leucocarpa var. yunnanensis (DBZP) alleviates rheumatoid arthritis through ameliorating gut microbiota. Int J Biol Macromol. (2024) 281:136250. doi: 10.1016/j.ijbiomac.2024.136250, PMID: 39482128

[78] Gou Y Zhang J Li C Liu Y Hui J Zhou R. Causal relationship between gut microbiota and rheumatoid arthritis: a two-sample Mendelian randomisation study. Clin Exp Rheumatol. (2024) 42:166–73. doi: 10.55563/clinexprheumatol/p9ig7c, PMID: 37812479

[79] Yu D Du J Pu X Zheng L Chen S Wang N. The gut microbiome and metabolites are altered and interrelated in patients with rheumatoid arthritis. Front Cell Infect Microbiol. (2021) 11:763507. doi: 10.3389/fcimb.2021.763507, PMID: 35145919 PMC8821809

[80] Catrina AI Deane KD Scher JU. Gene, environment, microbiome and mucosal immune tolerance in rheumatoid arthritis. Rheumatol (Oxford). (2016) 55:391–402. doi: 10.1093/rheumatology/keu469, PMID: 25539828 PMC4746430

[81] Cheng J Hu J Geng F Nie S. Bacteroides utilization for dietary polysaccharides and their beneficial effects on gut health. Food Sci Hum Wellness. (2022) 11:1101–10. doi: 10.1016/j.fshw.2022.04.002

[82] Brandl C Bucci L Schett G Zaiss MM. Crossing the barriers: Revisiting the gut feeling in rheumatoid arthritis. Eur J Immunol. (2021) 51:798–810. doi: 10.1002/eji.202048876, PMID: 33594693

[83] Yang W Cong Y. Gut microbiota-derived metabolites in the regulation of host immune responses and immune-related inflammatory diseases. Cell Mol Immunol. (2021) 18:866–77. doi: 10.1038/s41423-021-00661-4, PMID: 33707689 PMC8115644

[84] Zhao Y Cheng M Zou L Yin L Zhong C Zha Y. Hidden link in gut-joint axis: gut microbes promote rheumatoid arthritis at early stage by enhancing ascorbate degradation. Gut. (2022) 71:1041–43. doi: 10.1136/gutjnl-2021-325209, PMID: 34244347 PMC8995803

[85] Zheng D Liwinski T Elinav E. Interaction between microbiota and immunity in health and disease. Cell Res. (2020) 30:492–506. doi: 10.1038/s41422-020-0332-7, PMID: 32433595 PMC7264227

[86] Parada Venegas D de la Fuente MK Landskron G Gonzalez MJ Quera R Dijkstra G. Short chain fatty acids (SCFAs)-mediated gut epithelial and immune regulation and its relevance for inflammatory bowel diseases. Front Immunol. (2019) 10:277. doi: 10.3389/fimmu.2019.00277, PMID: 30915065 PMC6421268

[87] Mowat AM Agace WW. Regional specialization within the intestinal immune system. Nat Rev Immunol. (2014) 14:667–85. doi: 10.1038/nri3738, PMID: 25234148

[88] Maynard CL Harrington LE Janowski KM Oliver JR Zindl CL Rudensky AY. Regulatory T cells expressing interleukin 10 develop from Foxp3+ and Foxp3- precursor cells in the absence of interleukin 10. Nat Immunol. (2007) 8:931–41. doi: 10.1038/ni1504, PMID: 17694059

[89] Hadis U Wahl B Schulz O Hardtke-Wolenski M Schippers A Wagner N. Intestinal tolerance requires gut homing and expansion of FoxP3+ regulatory T cells in the lamina propria. Immunity. (2011) 34:237–46. doi: 10.1016/j.immuni.2011.01.016, PMID: 21333554

[90] Schiering C Krausgruber T Chomka A Frohlich A Adelmann K Wohlfert EA. The alarmin IL-33 promotes regulatory T-cell function in the intestine. Nature. (2014) 513:564–68. doi: 10.1038/nature13577, PMID: 25043027 PMC4339042

[91] Harrison OJ Srinivasan N Pott J Schiering C Krausgruber T Ilott NE. Epithelial-derived IL-18 regulates Th17 cell differentiation and Foxp3(+) Treg cell function in the intestine. Mucosal Immunol. (2015) 8:1226–36. doi: 10.1038/mi.2015.13, PMID: 25736457 PMC4368110

[92] Shan M Gentile M Yeiser JR Walland AC Bornstein VU Chen K. Mucus enhances gut homeostasis and oral tolerance by delivering immunoregulatory signals. Science. (2013) 342:447–53. doi: 10.1126/science.1237910, PMID: 24072822 PMC4005805

[93] Coombes JL Siddiqui KR Arancibia-Cárcamo CV Hall J Sun CM Belkaid Y. A functionally specialized population of mucosal CD103+ DCs induces Foxp3+ regulatory T cells via a TGF-beta and retinoic acid-dependent mechanism. J Exp Med. (2007) 204:1757–64. doi: 10.1084/jem.20070590, PMID: 17620361 PMC2118683

[94] Ishigame H Zenewicz LA Sanjabi S Licona-Limón P Nakayama M Leonard WJ. Excessive Th1 responses due to the absence of TGF-β signaling cause autoimmune diabetes and dysregulated Treg cell homeostasis. Proc Natl Acad Sci USA. (2013) 110:6961–66. doi: 10.1073/pnas.1304498110, PMID: 23569233 PMC3637710

[95] Smith PM Howitt MR Panikov N Michaud M Gallini CA Bohlooly-Y M. The microbial metabolites, short-chain fatty acids, regulate colonic Treg cell homeostasis. Science. (2013) 341:569–73. doi: 10.1126/science.1241165, PMID: 23828891 PMC3807819

[96] Tang Y Chen C Jiang B Wang L Jiang F Wang D. Bifidobacterium bifidum-mediated specific delivery of nanoparticles for tumor therapy. Int J Nanomedicine. (2021) 16:4643–59. doi: 10.2147/IJN.S315650, PMID: 34267516 PMC8275162

[97] Jeong Y Jhun J Lee S Na HS Choi J Cho K. Therapeutic potential of a novel bifidobacterium identified through microbiome profiling of RA patients with different RF levels. Front Immunol. (2021) 12:736196. doi: 10.3389/fimmu.2021.736196, PMID: 34867956 PMC8634832

[98] Yang Y Hong Q Zhang X Liu Z. Bifidobacterium animalis BD400 protects from collagen-induced arthritis through histidine metabolism. Front Immunol. (2025) 16:1518181. doi: 10.3389/fimmu.2025.1518181, PMID: 39911381 PMC11794514

[99] Sun S Luo L Liang W Yin Q Guo J Rush AM. Bifidobacterium alters the gut microbiota and modulates the functional metabolism of T regulatory cells in the context of immune checkpoint blockade. Proc Natl Acad Sci U.S.A. (2020) 117:27509–15. doi: 10.1073/pnas.1921223117, PMID: 33077598 PMC7959554

[100] Verma R Lee C Jeun E Yi J Kim KS Ghosh A. Cell surface polysaccharides of Bifidobacterium bifidum induce the generation of Foxp3(+) regulatory T cells. Sci Immunol. (2018) 3:eaat6975. doi: 10.1126/sciimmunol.aat6975, PMID: 30341145

[101] Li B Ding M Liu X Zhao J Ross RP Stanton C. Bifidobacterium breve CCFM1078 Alleviates Collagen-Induced Arthritis in Rats via Modulating the Gut Microbiota and Repairing the Intestinal Barrier Damage. J Agric Food Chem. (2022) 70:14665–78. doi: 10.1021/acs.jafc.2c04602, PMID: 36377740

[102] Li D Cheng J Zhu Z Catalfamo M Goerlitz D Lawless OJ. Treg-inducing capacity of genomic DNA of Bifidobacterium longum subsp. infantis. Allergy Asthma Proc. (2020) 41:372–85. doi: 10.2500/aap.2020.41.200064, PMID: 32867892 PMC8242987

[103] Li D Cruz I Sorkhabi S Foley PL Wagner J Bellanti JA. Dose-response studies of methylated and nonmethylated CpG ODNs from Bifidobacterium longum subsp. infantis optimizing Treg Cell stimulation. Allergy Asthma Proc. (2025) 46:98–104. doi: 10.2500/aap.2025.46.250001, PMID: 40011992 PMC13491394

[104] Miri ST Sotoodehnejadnematalahi F Amiri MM Pourshafie MR Rohani M. The impact of Lactobacillus and Bifidobacterium probiotic cocktail on modulation of gene expression of gap junctions dysregulated by intestinal pathogens. Arch Microbiol. (2022) 204:417. doi: 10.1007/s00203-022-03026-1, PMID: 35737111

[105] Zhao Q Ren H Yang N Xia X Chen Q Zhou D. Bifidobacterium pseudocatenulatum-Mediated Bile Acid Metabolism to Prevent Rheumatoid Arthritis via the Gut-Joint Axis. Nutrients. (2023) 15:255. doi: 10.3390/nu15020255, PMID: 36678126 PMC9861548

[106] Corcoran BM Stanton C Fitzgerald GF Ross RP. Survival of probiotic lactobacilli in acidic environments is enhanced in the presence of metabolizable sugars. Appl Environ Microbiol. (2005) 71:3060–67. doi: 10.1128/AEM.71.6.3060-3067.2005, PMID: 15933002 PMC1151822

[107] Bungau SG Behl T Singh A Sehgal A Singh S Chigurupati S. Targeting probiotics in rheumatoid arthritis. Nutrients. (2021) 13:3376. doi: 10.3390/nu13103376, PMID: 34684377 PMC8539185

[108] Paul AK Paul A Jahan R Jannat K Bondhon TA Hasan A. Probiotics and Amelioration of Rheumatoid Arthritis: Significant Roles of Lactobacillus casei and Lactobacillus acidophilus. Microorganisms. (2021) 9:1070. doi: 10.3390/microorganisms9051070, PMID: 34065638 PMC8157104

[109] Amdekar S Singh V Kumar A Sharma P Singh R. Lactobacillus casei and Lactobacillus acidophilus regulate inflammatory pathway and improve antioxidant status in collagen-induced arthritic rats. J Interferon Cytokine Res. (2013) 33:1–08. doi: 10.1089/jir.2012.0034, PMID: 23030670

[110] Fan Z Ross RP Stanton C Hou B Zhao J Zhang H. Lactobacillus casei CCFM1074 Alleviates Collagen-Induced Arthritis in Rats via Balancing Treg/Th17 and Modulating the Metabolites and Gut Microbiota. Front Immunol. (2021) 12:680073. doi: 10.3389/fimmu.2021.680073, PMID: 34079556 PMC8165437

[111] Pan H Guo R Ju Y Wang Q Zhu J Xie Y. A single bacterium restores the microbiome dysbiosis to protect bones from destruction in a rat model of rheumatoid arthritis. Microbiome. (2019) 7:107. doi: 10.1186/s40168-019-0719-1, PMID: 31315667 PMC6637628

[112] Mikulic J Longet S Favre L Benyacoub J Corthesy B. Secretory IgA in complex with Lactobacillus rhamnosus potentiates mucosal dendritic cell-mediated Treg cell differentiation via TLR regulatory proteins, RALDH2 and secretion of IL-10 and TGF-beta. Cell Mol Immunol. (2017) 14:546–56. doi: 10.1038/cmi.2015.110, PMID: 26972771 PMC5518813

[113] Teng X Mou D Li H Jiao L Wu S Pi J. SIGIRR deficiency contributes to CD4 T cell abnormalities by facilitating the IL1/C/EBPbeta/TNF-alpha signaling axis in rheumatoid arthritis. Mol Med. (2022) 28:135. doi: 10.1186/s10020-022-00563-9, PMID: 36401167 PMC9673409

[114] Mucida D Pino-Lagos K Kim G Nowak E Benson MJ Kronenberg M. Retinoic acid can directly promote TGF-beta-mediated Foxp3(+) Treg cell conversion of naive T cells. Immunity. (2009) 30:471–72. doi: 10.1016/j.immuni.2009.03.008, PMID: 19371709 PMC2864308

[115] Liu X Zhang J Zou Q Zhong B Wang H Mou F. Lactobacillus salivarius isolated from patients with rheumatoid arthritis suppresses collagen-induced arthritis and increases treg frequency in mice. J Interferon Cytokine Res. (2016) 36:706–12. doi: 10.1089/jir.2016.0057, PMID: 27845855

[116] Zhai Q Shen X Cen S Zhang C Tian F Zhao J. Screening of Lactobacillus salivarius strains from the feces of Chinese populations and the evaluation of their effects against intestinal inflammation in mice. Food Funct. (2020) 11:221–35. doi: 10.1039/c9fo02116g, PMID: 31915776

[117] Zhou B Dong C Zhao B Lin K Tian Y Zhang R. Bacteroides fragilis participates in the therapeutic effect of methotrexate on arthritis through metabolite regulation. Front Microbiol. (2022) 13:1015130. doi: 10.3389/fmicb.2022.1015130, PMID: 36590441 PMC9798205

[118] Round JL Mazmanian SK. Inducible Foxp3+ regulatory T-cell development by a commensal bacterium of the intestinal microbiota. Proc Natl Acad Sci U.S.A. (2010) 107:12204–09. doi: 10.1073/pnas.0909122107, PMID: 20566854 PMC2901479

[119] Telesford KM Yan W Ochoa-Reparaz J Pant A Kircher C Christy MA. A commensal symbiotic factor derived from Bacteroides fragilis promotes human CD39(+)Foxp3(+) T cells and Treg function. Gut Microbes. (2015) 6:234–42. doi: 10.1080/19490976.2015.1056973, PMID: 26230152 PMC4615798

[120] Bodkhe R Balakrishnan B Taneja V. The role of microbiome in rheumatoid arthritis treatment. Ther Adv Musculoskelet Dis. (2019) 11:1759720X19844632. doi: 10.1177/1759720X19844632, PMID: 31431810 PMC6685117

[121] Fitzgerald CB Shkoporov AN Sutton TDS Chaplin AV Velayudhan V Ross RP. Comparative analysis of Faecalibacterium prausnitzii genomes shows a high level of genome plasticity and warrants separation into new species-level taxa. BMC Genomics. (2018) 19:931. doi: 10.1186/s12864-018-5313-6, PMID: 30547746 PMC6295017

[122] Moon J Lee AR Kim H Jhun J Lee S Choi JW. Faecalibacterium prausnitzii alleviates inflammatory arthritis and regulates IL-17 production, short chain fatty acids, and the intestinal microbial flora in experimental mouse model for rheumatoid arthritis. Arthritis Res Ther. (2023) 25:130. doi: 10.1186/s13075-023-03118-3, PMID: 37496081 PMC10373287

[123] Jotereau F Alameddine J Teusan R Pedron A Jouand N Altare F. Human gut microbiota-reactive DP8alpha regulatory T cells, signature and related emerging functions. Front Immunol. (2022) 13:1026994. doi: 10.3389/fimmu.2022.1026994, PMID: 36479125 PMC9720269

[124] Neumann C Blume J Roy U Teh PP Vasanthakumar A Beller A. c-Maf-dependent T(reg) cell control of intestinal T(H)17 cells and IgA establishes host-microbiota homeostasis. Nat Immunol. (2019) 20:471–81. doi: 10.1038/s41590-019-0316-2, PMID: 30778241

[125] Sarrabayrouse G Bossard C Chauvin JM Jarry A Meurette G Quévrain E. CD4CD8αα lymphocytes, a novel human regulatory T cell subset induced by colonic bacteria and deficient in patients with inflammatory bowel disease. PLoS Biol. (2014) 12:e1001833. doi: 10.1371/journal.pbio.1001833, PMID: 24714093 PMC3979654

[126] Alameddine J Godefroy E Papargyris L Sarrabayrouse G Tabiasco J Bridonneau C. Faecalibacterium prausnitzii Skews Human DC to Prime IL10-Producing T Cells Through TLR2/6/JNK Signaling and IL-10, IL-27, CD39, and IDO-1 Induction. Front Immunol. (2019) 10:143. doi: 10.3389/fimmu.2019.00143, PMID: 30787928 PMC6373781

[127] Alpizar-Rodriguez D Lesker TR Gronow A Gilbert B Raemy E Lamacchia C. Prevotella copri in individuals at risk for rheumatoid arthritis. Ann Rheum Dis. (2019) 78:590–93. doi: 10.1136/annrheumdis-2018-214514, PMID: 30760471

[128] Jiang L Shang M Yu S Liu Y Zhang H Zhou Y. A high-fiber diet synergizes with Prevotella copri and exacerbates rheumatoid arthritis. Cell Mol Immunol. (2022) 19:1414–24. doi: 10.1038/s41423-022-00934-6, PMID: 36323929 PMC9709035

[129] Bodkhe R Marietta EV Balakrishnan B Luckey DH Horwath IE Shouche YS. Human gut-derived commensal suppresses generation of T-cell response to gliadin in humanized mice by modulating gut microbiota. Anaerobe. (2021) 68:102237. doi: 10.1016/j.anaerobe.2020.102237, PMID: 32721554

[130] Bianchini R Bistoni O Alunno A Petrillo MG Ronchetti S Sportoletti P. CD4(+) CD25(low) GITR(+) cells: a novel human CD4(+) T-cell population with regulatory activity. Eur J Immunol. (2011) 41:2269–78. doi: 10.1002/eji.201040943, PMID: 21557210

[131] Balakrishnan B Johnson S Luckey D Marietta E Murray J Taneja V. Small intestinal derived Prevotella histicola simulates biologic as a therapeutic agent. Sci Rep. (2024) 14:29217. doi: 10.1038/s41598-024-80635-4, PMID: 39587228 PMC11589831

[132] Kim S Seo S Kweon M. Gut microbiota-derived metabolites tune host homeostasis fate. Semin Immunopathol. (2024) 46:2. doi: 10.1007/s00281-024-01012-x, PMID: 38990345 PMC11239740

[133] Becattini S Sorbara MT Kim SG Littmann EL Dong Q Walsh G. Rapid transcriptional and metabolic adaptation of intestinal microbes to host immune activation. Cell Host Microbe. (2021) 29:378–93. doi: 10.1016/j.chom.2021.01.003, PMID: 33539766 PMC7954923

[134] Cai X Ren F Yao Y. Gut microbiota and their metabolites in the immune response of rheumatoid arthritis: Therapeutic potential and future directions. Int Immunopharmacol. (2025) 147:114034. doi: 10.1016/j.intimp.2025.114034, PMID: 39805176

[135] Singh V Lee G Son H Koh H Kim ES Unno T. Butyrate producers, “The Sentinel of Gut”: Their intestinal significance with and beyond butyrate, and prospective use as microbial therapeutics. Front Microbiol. (2022) 13:1103836. doi: 10.3389/fmicb.2022.1103836, PMID: 36713166 PMC9877435

[136] Makki K Deehan EC Walter J Backhed F. The impact of dietary fiber on gut microbiota in host health and disease. Cell Host Microbe. (2018) 23:705–15. doi: 10.1016/j.chom.2018.05.012, PMID: 29902436

[137] Koh A De Vadder F Kovatcheva-Datchary P Backhed F. From dietary fiber to host physiology: short-chain fatty acids as key bacterial metabolites. Cell. (2016) 165:1332–45. doi: 10.1016/j.cell.2016.05.041, PMID: 27259147

[138] Wang L Liu J. Localized butyrate restores gut homeostasis. Nat BioMed Eng. (2023) 7:3–05. doi: 10.1038/s41551-022-00994-z, PMID: 36550306

[139] Thomas SP Denu JM. Short-chain fatty acids activate acetyltransferase p300. Elife. (2021) 10:e72171. doi: 10.7554/eLife.72171, PMID: 34677127 PMC8585482

[140] Furusawa Y Obata Y Fukuda S Endo TA Nakato G Takahashi D. Commensal microbe-derived butyrate induces the differentiation of colonic regulatory T cells. Nature. (2013) 504:446–50. doi: 10.1038/nature12721, PMID: 24226770

[141] Arpaia N Campbell C Fan X Dikiy S van der Veeken J DeRoos P. Metabolites produced by commensal bacteria promote peripheral regulatory T-cell generation. Nature. (2013) 504:451–55. doi: 10.1038/nature12726, PMID: 24226773 PMC3869884

[142] Kim DS Kwon J Lee SH Kim EK Ryu J Jung K. Attenuation of rheumatoid inflammation by sodium butyrate through reciprocal targeting of HDAC2 in osteoclasts and HDAC8 in T cells. Front Immunol. (2018) 9:1525. doi: 10.3389/fimmu.2018.01525, PMID: 30034392 PMC6043689

[143] Takahashi D Hoshina N Kabumoto Y Maeda Y Suzuki A Tanabe H. Microbiota-derived butyrate limits the autoimmune response by promoting the differentiation of follicular regulatory T cells. EBioMedicine. (2020) 58:102913. doi: 10.1016/j.ebiom.2020.102913, PMID: 32711255 PMC7387783

[144] Sun M Wu W Liu Z Cong Y. Microbiota metabolite short chain fatty acids, GPCR, and inflammatory bowel diseases. J Gastroenterol. (2017) 52:1–08. doi: 10.1007/s00535-016-1242-9, PMID: 27448578 PMC5215992

[145] Huang S Hu S Liu S Tang B Liu Y Tang L. Lithium carbonate alleviates colon inflammation through modulating gut microbiota and Treg cells in a GPR43-dependent manner. Pharmacol Res. (2022) 175:105992. doi: 10.1016/j.phrs.2021.105992, PMID: 34801681

[146] Schilderink R Verseijden C Seppen J Muncan V van den Brink GR Lambers TT. The SCFA butyrate stimulates the epithelial production of retinoic acid via inhibition of epithelial HDAC. Am J Physiol Gastrointest Liver Physiol. (2016) 310:G1138–46. doi: 10.1152/ajpgi.00411.2015, PMID: 27151945

[147] Graham KL Werner BJ Moyer KM Patton AK Krois CR Yoo HS. DGAT1 inhibits retinol-dependent regulatory T cell formation and mediates autoimmune encephalomyelitis. Proc Natl Acad Sci U.S.A. (2019) 116:3126–35. doi: 10.1073/pnas.1817669116, PMID: 30718413 PMC6386656

[148] Raverdeau M Christofi M Malara A Wilk MM Misiak A Kuffova L. Retinoic acid-induced autoantigen-specific type 1 regulatory T cells suppress autoimmunity. EMBO Rep. (2019) 20:e47121. doi: 10.15252/embr.201847121, PMID: 30894405 PMC6500997

[149] Gurav A Sivaprakasam S Bhutia YD Boettger T Singh N Ganapathy V. Slc5a8, a Na+-coupled high-affinity transporter for short-chain fatty acids, is a conditional tumour suppressor in colon that protects against colitis and colon cancer under low-fibre dietary conditions. Biochem J. (2015) 469:267–78. doi: 10.1042/BJ20150242, PMID: 25984582 PMC4943859

[150] Kim CH. Complex regulatory effects of gut microbial short-chain fatty acids on immune tolerance and autoimmunity. Cell Mol Immunol. (2023) 20:341–50. doi: 10.1038/s41423-023-00987-1, PMID: 36854801 PMC10066346

[151] Bai Y Li Y Marion T Tong Y Zaiss MM Tang Z. Resistant starch intake alleviates collagen-induced arthritis in mice by modulating gut microbiota and promoting concomitant propionate production. J Autoimmun. (2021) 116:102564. doi: 10.1016/j.jaut.2020.102564, PMID: 33203617

[152] Hu M Eviston D Hsu P Marino E Chidgey A Santner-Nanan B. Decreased maternal serum acetate and impaired fetal thymic and regulatory T cell development in preeclampsia. Nat Commun. (2019) 10:3031. doi: 10.1038/s41467-019-10703-1, PMID: 31292453 PMC6620275

[153] Jones BV Begley M Hill C Gahan CGM Marchesi JR. Functional and comparative metagenomic analysis of bile salt hydrolase activity in the human gut microbiome. Proc Natl Acad Sci U.S.A. (2008) 105:13580–85. doi: 10.1073/pnas.0804437105, PMID: 18757757 PMC2533232

[154] Song Z Cai Y Lao X Wang X Lin X Cui Y. Taxonomic profiling and populational patterns of bacterial bile salt hydrolase (BSH) genes based on worldwide human gut microbiome. Microbiome. (2019) 7:9. doi: 10.1186/s40168-019-0628-3, PMID: 30674356 PMC6345003

[155] Lavelle A Sokol H. Gut microbiota-derived metabolites as key actors in inflammatory bowel disease. Nat Rev Gastroenterol Hepatol. (2020) 17:223–37. doi: 10.1038/s41575-019-0258-z, PMID: 32076145

[156] Li W Hang S Fang Y Bae S Zhang Y Zhang M. A bacterial bile acid metabolite modulates T(reg) activity through the nuclear hormone receptor NR4A1. Cell Host Microbe. (2021) 29:1366–77. doi: 10.1016/j.chom.2021.07.013, PMID: 34416161 PMC9064000

[157] Song X Sun X Oh SF Wu M Zhang Y Zheng W. Microbial bile acid metabolites modulate gut RORgamma(+) regulatory T cell homeostasis. Nature. (2020) 577:410–15. doi: 10.1038/s41586-019-1865-0, PMID: 31875848 PMC7274525

[158] Campbell C McKenney PT Konstantinovsky D Isaeva OI Schizas M Verter J. Bacterial metabolism of bile acids promotes generation of peripheral regulatory T cells. Nature. (2020) 581:475–79. doi: 10.1038/s41586-020-2193-0, PMID: 32461639 PMC7540721

[159] Liu J Peng F Cheng H Zhang D Zhang Y Wang L. Chronic cold environment regulates rheumatoid arthritis through modulation of gut microbiota-derived bile acids. Sci Total Environ. (2023) 903:166837. doi: 10.1016/j.scitotenv.2023.166837, PMID: 37689184

[160] Zhang Y Gao X Gao S Liu Y Wang W Feng Y. Effect of gut flora mediated-bile acid metabolism on intestinal immune microenvironment. Immunology. (2023) 170:301–18. doi: 10.1111/imm.13672, PMID: 37317655

[161] Hang S Paik D Yao L Kim E Trinath J Lu J. Bile acid metabolites control T H 17 and T reg cell differentiation. Nature. (2019) 576:143–48. doi: 10.1038/s41586-019-1785-z, PMID: 31776512 PMC6949019

[162] Moulin D Millard M Taieb M Michaudel C Aucouturier A Lefevre A. Counteracting tryptophan metabolism alterations as a new therapeutic strategy for rheumatoid arthritis. Ann Rheum Dis. (2024) 83:312–23. doi: 10.1136/ard-2023-224014, PMID: 38049981 PMC10894831

[163] Opitz CA Litzenburger UM Sahm F Ott M Tritschler I Trump S. An endogenous tumour-promoting ligand of the human aryl hydrocarbon receptor. Nature. (2011) 478:197–203. doi: 10.1038/nature10491, PMID: 21976023

[164] Mezrich JD Fechner JH Zhang X Johnson BP Burlingham WJ Bradfield CA. An interaction between kynurenine and the aryl hydrocarbon receptor can generate regulatory T cells. J Immunol. (2010) 185:3190–98. doi: 10.4049/jimmunol.0903670, PMID: 20720200 PMC2952546

[165] Solvay M Holfelder P Klaessens S Pilotte L Stroobant V Lamy J. Tryptophan depletion sensitizes the AHR pathway by increasing AHR expression and GCN2/LAT1-mediated kynurenine uptake, and potentiates induction of regulatory T lymphocytes. J Immunother Cancer. (2023) 11:e006728. doi: 10.1136/jitc-2023-006728, PMID: 37344101 PMC10314700

[166] Agus A Planchais J Sokol H. Gut microbiota regulation of tryptophan metabolism in health and disease. Cell Host Microbe. (2018) 23:716–24. doi: 10.1016/j.chom.2018.05.003, PMID: 29902437

[167] Jiang Z Zeng S Huang T Lin Y Wang F Gao X. Sinomenine ameliorates rheumatoid arthritis by modulating tryptophan metabolism and activating aryl hydrocarbon receptor via gut microbiota regulation. Sci Bull (Beijing). (2023) 68:1540–55. doi: 10.1016/j.scib.2023.06.027, PMID: 37422372

[168] Su X Wang X Zhang X Sun Y Jia Y. beta-Indole-3-acetic acid attenuated collagen-induced arthritis through reducing the ubiquitination of Foxp3 via the AhR-TAZ-Tip60 pathway. Immunol Res. (2024) 72:741–53. doi: 10.1007/s12026-024-09480-x, PMID: 38630408

[169] Nakamura A Matsumoto M. Role of polyamines in intestinal mucosal barrier function. Semin Immunopathol. (2025) 47:9. doi: 10.1007/s00281-024-01035-4, PMID: 39836273 PMC11750915

[170] Cheng Q Ni L Liu A Huang X Xiang P Zhang Q. Spermidine protects cartilage from IL-1beta-mediated ferroptosis. Mol Cell Biochem. (2024) 479:2785–94. doi: 10.1007/s11010-023-04889-8, PMID: 38040913

[171] Carriche GM Almeida L Stuve P Velasquez L Dhillon-LaBrooy A Roy U. Regulating T-cell differentiation through the polyamine spermidine. J Allergy Clin Immunol. (2021) 147:335–48. doi: 10.1016/j.jaci.2020.04.037, PMID: 32407834

[172] Yang R Qu C Zhou Y Konkel JE Shi S Liu Y. Hydrogen sulfide promotes tet1- and tet2-mediated foxp3 demethylation to drive regulatory T cell differentiation and maintain immune homeostasis. Immunity. (2015) 43:251–63. doi: 10.1016/j.immuni.2015.07.017, PMID: 26275994 PMC4731232

[173] Coyte KZ Rakoff-Nahoum S. Understanding competition and cooperation within the mammalian gut microbiome. Curr Biol. (2019) 29:R538–44. doi: 10.1016/j.cub.2019.04.017, PMID: 31163167 PMC6935513

[174] Cannarella LAT Mari NL Alcantara CC Iryioda TMV Costa NT Oliveira SR. Mixture of probiotics reduces inflammatory biomarkers and improves the oxidative/nitrosative profile in people with rheumatoid arthritis. Nutrition. (2021) 89:111282. doi: 10.1016/j.nut.2021.111282, PMID: 34111674

[175] Zamani B Farshbaf S Golkar HR Bahmani F Asemi Z. Synbiotic supplementation and the effects on clinical and metabolic responses in patients with rheumatoid arthritis: a randomised, double-blind, placebo-controlled trial - Expression of concern. Br J Nutr. (2022) 127:156. doi: 10.1017/S0007114521002051, PMID: 34353393

[176] Zamani B Golkar HR Farshbaf S Emadi-Baygi M Tajabadi-Ebrahimi M Jafari P. Clinical and metabolic response to probiotic supplementation in patients with rheumatoid arthritis: a randomized, double-blind, placebo-controlled trial. Int J Rheum Dis. (2016) 19:869–79. doi: 10.1111/1756-185X.12888, PMID: 27135916

[177] Yang Y Hong Q Zhang X Liu Z. Protective effect of a combination of multiple strains of Lactobacillus acidophilus on collagen-induced arthritis. Food Funct. (2025) 16:943–65. doi: 10.1039/d4fo05273k, PMID: 39807088

[178] Kwon H Lee C So J Chae C Hwang J Sahoo A. Generation of regulatory dendritic cells and CD4+Foxp3+ T cells by probiotics administration suppresses immune disorders. Proc Natl Acad Sci U.S.A. (2010) 107:2159–64. doi: 10.1073/pnas.0904055107, PMID: 20080669 PMC2836639

[179] Zeng H Umar S Rust B Lazarova D Bordonaro M. Secondary bile acids and short chain fatty acids in the colon: A focus on colonic microbiome, cell proliferation, inflammation, and cancer. Int J Mol Sci. (2019) 20:1214. doi: 10.3390/ijms20051214, PMID: 30862015 PMC6429521

[180] Kolodziejczyk AA Zheng D Elinav E. Diet-microbiota interactions and personalized nutrition. Nat Rev Microbiol. (2019) 17:742–53. doi: 10.1038/s41579-019-0256-8, PMID: 31541197

[181] Ma F Li Z Liu H Chen S Zheng S Zhu J. Dietary-timing-induced gut microbiota diurnal oscillations modulate inflammatory rhythms in rheumatoid arthritis. Cell Metab. (2024) 36:2367–82. doi: 10.1016/j.cmet.2024.08.007, PMID: 39260371

[182] Laragione T Harris C Azizgolshani N Beeton C Bongers G Gulko PS. Magnesium increases numbers of Foxp3+ Treg cells and reduces arthritis severity and joint damage in an IL-10-dependent manner mediated by the intestinal microbiome. EBioMedicine. (2023) 92:104603. doi: 10.1016/j.ebiom.2023.104603, PMID: 37201335 PMC10203746

[183] Collins SL Stine JG Bisanz JE Okafor CD Patterson AD. Bile acids and the gut microbiota: metabolic interactions and impacts on disease. Nat Rev Microbiol. (2023) 21:236–47. doi: 10.1038/s41579-022-00805-x, PMID: 36253479 PMC12536349

[184] Nikiphorou E Philippou E. Nutrition and its role in prevention and management of rheumatoid arthritis. Autoimmun Rev. (2023) 22:103333. doi: 10.1016/j.autrev.2023.103333, PMID: 37182439

[185] Dourado E Ferro M Sousa Guerreiro C Fonseca JE. Diet as a modulator of intestinal microbiota in rheumatoid arthritis. Nutrients. (2020) 12:3504. doi: 10.3390/nu12113504, PMID: 33202579 PMC7696404

[186] Boytar AN Skinner TL Wallen RE Jenkins DG Dekker Nitert M. The effect of exercise prescription on the human gut microbiota and comparison between clinical and apparently healthy populations: A systematic review. Nutrients. (2023) 15:1534. doi: 10.3390/nu15061534, PMID: 36986264 PMC10054511

[187] Matenchuk BA Mandhane PJ Kozyrskyj AL. Sleep, circadian rhythm, and gut microbiota. Sleep Med Rev. (2020) 53:101340. doi: 10.1016/j.smrv.2020.101340, PMID: 32668369

[188] Galley JD Nelson MC Yu Z Dowd SE Walter J Kumar PS. Exposure to a social stressor disrupts the community structure of the colonic mucosa-associated microbiota. BMC Microbiol. (2014) 14:189. doi: 10.1186/1471-2180-14-189, PMID: 25028050 PMC4105248

[189] Wang R Li S Jin L Zhang W Liu N Wang H. Four-week administration of nicotinemoderately impacts blood metabolic profile and gut microbiota in a diet-dependent manner. BioMed Pharmacother. (2019) 115:108945. doi: 10.1016/j.biopha.2019.108945, PMID: 31100541

[190] M C C C N L L C M F J L G D A C G. Comparing the effects of acute alcohol consumption in germ-free and conventional mice: the role of the gut microbiota. BMC Microbiol. (2014) 14:240. doi: 10.1186/s12866-014-0240-4, PMID: 25223989 PMC4177591

[191] Zaragoza-Garcia O Castro-Alarcon N Perez-Rubio G Guzman-Guzman IP. DMARDs-gut microbiota feedback: implications in the response to therapy. Biomolecules. (2020) 10:1479. doi: 10.3390/biom10111479, PMID: 33114390 PMC7692063

[192] Yan H Su R Xue H Gao C Li X Wang C. Pharmacomicrobiology of methotrexate in rheumatoid arthritis: gut microbiome as predictor of therapeutic response. Front Immunol. (2021) 12:789334. doi: 10.3389/fimmu.2021.789334, PMID: 34975886 PMC8719371

[193] Heydari-Kamjani M Demory Beckler M Kesselman MM. Reconsidering the use of minocycline in the preliminary treatment regime of rheumatoid arthritis. Cureus. (2019) 11:e5351. doi: 10.7759/cureus.5351, PMID: 31608186 PMC6783212

[194] Ramirez J Guarner F Bustos Fernandez L Maruy A Sdepanian VL Cohen H. Antibiotics as major disruptors of gut microbiota. Front Cell Infect Microbiol. (2020) 10:572912. doi: 10.3389/fcimb.2020.572912, PMID: 33330122 PMC7732679

[195] Elias AJ Barna V Patoni C Demeter D Veres DS Bunduc S. Probiotic supplementation during antibiotic treatment is unjustified in maintaining the gut microbiome diversity: a systematic review and meta-analysis. BMC Med. (2023) 21:262. doi: 10.1186/s12916-023-02961-0, PMID: 37468916 PMC10355080

[196] Liang Y Liu M Cheng Y Wang X Wang W. Prevention and treatment of rheumatoid arthritis through traditional Chinese medicine: role of the gut microbiota. Front Immunol. (2023) 14:1233994. doi: 10.3389/fimmu.2023.1233994, PMID: 37781405 PMC10538529

[197] Qiao S Lian X Yue M Zhang Q Wei Z Chen L. Regulation of gut microbiota substantially contributes to the induction of intestinal Treg cells and consequent anti-arthritis effect of madecassoside. Int Immunopharmacol. (2020) 89:107047. doi: 10.1016/j.intimp.2020.107047, PMID: 33039960

[198] Peng J Lu X Xie K Xu Y He R Guo L. Dynamic alterations in the gut microbiota of collagen-induced arthritis rats following the prolonged administration of total glucosides of paeony. Front Cell Infect Microbiol. (2019) 9:204. doi: 10.3389/fcimb.2019.00204, PMID: 31245305 PMC6581682

[199] Liu J Zhang D Zhou Y Wu J Feng W Peng C. Fuzi alleviates cold-related rheumatoid arthritis via regulating gut microbiota and microbial bile acid metabolism. Chin Med. (2025) 20:64. doi: 10.1186/s13020-025-01123-z, PMID: 40375326 PMC12079872

[200] Wang N Li Y. Effects of acupuncture on intestinal microbiota in mice with rheumatoid arthritis. Zhen Ci Yan Jiu. (2024) 49:943–48. doi: 10.13702/j.1000-0607.20230307, PMID: 39401831

[201] Ajith TA Anita B. Impact of gut microbiota and probiotics on rheumatoid arthritis: A potential treatment challenge. Int J Rheum Dis. (2025) 28:e70266. doi: 10.1111/1756-185X.70266, PMID: 40329613

[202] Liu H Wang J He T Becker S Zhang G Li D. Butyrate: A double-edged sword for health? Adv Nutr. (2018) 9:21–9. doi: 10.1093/advances/nmx009, PMID: 29438462 PMC6333934

[203] Cao S Budina E Raczy MM Solanki A Nguyen M Beckman TN. A serine-conjugated butyrate prodrug with high oral bioavailability suppresses autoimmune arthritis and neuroinflammation in mice. Nat BioMed Eng. (2024) 8:611–27. doi: 10.1038/s41551-024-01190-x, PMID: 38561491 PMC11161413

